# Silver nanoparticles with excellent biocompatibility block pseudotyped SARS-CoV-2 in the presence of lung surfactant

**DOI:** 10.3389/fbioe.2022.1083232

**Published:** 2022-12-12

**Authors:** Govind Gupta, Bejan Hamawandi, Daniel J. Sheward, Ben Murrell, Leo Hanke, Gerald McInerney, Magda Blosi, Anna L. Costa, Muhammet S. Toprak, Bengt Fadeel

**Affiliations:** ^1^ Division of Molecular Toxicology, Institute of Environmental Medicine, Karolinska Institutet, Stockholm, Sweden; ^2^ Department of Applied Physics, KTH Royal Institute of Technology, Stockholm, Sweden; ^3^ Department of Microbiology, Tumor and Cell Biology, Karolinska Institutet, Stockholm, Sweden; ^4^ Institute of Science and Technology for Ceramics, National Research Council of Italy, Faenza, Italy

**Keywords:** human lung epithelium, nanoparticles, pseudovirus, silver, spike protein

## Abstract

Silver (Ag) is known to possess antimicrobial properties which is commonly attributed to soluble Ag ions. Here, we showed that Ag nanoparticles (NPs) potently inhibited SARS-CoV-2 infection using two different pseudovirus neutralization assays. We also evaluated a set of Ag nanoparticles of different sizes with varying surface properties, including polyvinylpyrrolidone (PVP)-coated and poly (ethylene glycol) (PEG)-modified Ag nanoparticles, and found that only the bare (unmodified) nanoparticles were able to prevent virus infection. For comparison, TiO_2_ nanoparticles failed to intercept the virus. Proteins and lipids may adsorb to nanoparticles forming a so-called bio-corona; however, Ag nanoparticles pre-incubated with pulmonary surfactant retained their ability to block virus infection in the present model. Furthermore, the secondary structure of the spike protein of SARS-CoV-2 was perturbed by the Ag nanoparticles, but not by the ionic control (AgNO_3_) nor by the TiO_2_ nanoparticles. Finally, Ag nanoparticles were shown to be non-cytotoxic towards the human lung epithelial cell line BEAS-2B and this was confirmed by using primary human nasal epithelial cells. These results further support that Ag nanoparticles may find use as anti-viral agents.

## 1 Introduction

The severe acute respiratory syndrome coronavirus 2 (SARS-CoV-2) caused a global health emergency, and safe and effective vaccinations are required to quell the current (and future) pandemics (Shin et al., 2020). However, while vaccines are important, there is also a need for approaches to control virus transmission in society at large and here biological agents may fall short due to their limited stability in harsh environments. Instead, non-biological disinfectants play an important role in controlling the spread of the virus (Talebian et al., 2020). Indeed, materials science may provide tools for the protection against and detection and treatment of SARS-CoV-2 infection beyond vaccines and anti-viral drug carriers (Tang et al., 2020). For example, several studies have shown that artificial or biological nanoparticles endowed with angiotensin converting enzyme II (ACE2) receptors or anti-SARS-CoV-2 neutralizing antibodies may act as decoys and inhibit the interaction of the virus with host cells (Rao et al., 2020; Zhang et al., 2020; Chen et al., 2021). However, these proof-of-principle approaches are far from being realized in the clinic. Indeed, these multi-component systems are complex, making scale-up a formidable challenge. By contrast, metallic nanoparticles (NPs), including cupric and cuprous oxide NPs, offer considerable advantages as disinfectants due to intrinsic antimicrobial properties including reactive oxygen species generation and photo-dynamic and/or photo-thermal capabilities (Behzadinasab et al., 2020; Hosseini et al., 2021; Merkl et al., 2021). Silver (Ag) NPs are also known to possess antimicrobial properties, and the antibacterial effects were suggested to depend both on the undissolved particles and on the leaching of soluble Ag (Faiz et al., 2018). Mechanistically, Ag NPs may exert a destabilizing effect on bacterial membranes (Gunawan et al., 2020). Furthermore, previous work has shown that Ag NPs can inhibit the infection of host cells with transmissible gastroenteritis virus, a porcine coronavirus, and the authors speculated that this could be due to an effect of Ag NPs on surface proteins of the virus (Lv et al., 2014). In a recent study, Ag NPs inhibited SARS-CoV-2 in a size-dependent manner, as evidenced using a pseudovirus assay (Jeremiah et al., 2020) (a pseudovirus is achieved by “pseudotyping” a non-replicating virus with outer membrane proteins or envelope proteins of the virus of interest, mimicking the infectious process of the live virus). The authors speculated that the NPs exerted their anti-viral effect by disrupting disulfide bonds on the spike protein of SARS-CoV-2 and/or its receptor (ACE2) on host cells, though evidence in support of this conjecture was not provided. In another recent study, He et al. (2022) evaluated a panel of Ag NPs with respect to their virucidal activity using an *in vitro* model based on the infection of Vero E6 cells with SARS-CoV-2. The authors found that the zeta potential of the surface-modified NPs correlated with the virus titer. Thus, several studies have suggested that metallic NPs or surfaces coated with such materials may act as disinfectants. However, the mechanism underlying these anti-viral effects is poorly understood. Moreover, it has not been determined whether the adsorbed bio-corona of host-derived proteins or lipids on the surface of the NPs could impede or otherwise modulate the anti-viral activities of Ag NPs. Notably, while the so-called protein corona on NPs has been widely studied (Walkey et al., 2014), it is also important to consider the potential role of the pulmonary surfactant (protein and lipid) derived corona (Hu et al., 2017). Finally, close attention should be paid to whether any novel anti-viral NPs are safe for humans or whether they could elicit cytotoxicity towards host cells. Here, we examined a set of Ag NPs with varying surface properties in the presence or absence of lung surfactant with respect to anti-viral effects using *in vitro* assays. The NPs were also evaluated for their biocompatibility using a human lung epithelial cell line and primary nasal epithelial cells.

## 2 Materials and methods

### 2.1 Nanoparticles

Silver (Ag) nanoparticles (NPs) (cat No. 484059-5G) were procured from Sigma (Sweden) as a powder. Additionally, Ag NPs (citrate-stabilized) of different sizes (10, 50, and 100 nm) and varying surface properties (50 nm PVP-40 kDa and PEG-5 kDa Ag NPs) were purchased from NanoComposix, Inc. (San Diego, CA, United States). These NPs were all provided in colloidal form as stock solutions (1 mg/ml) in MilliQ^®^ water (PVP-coated) or aqueous 2 mM citrate solutions (for citrate-stabilized NPs). Titanium dioxide (TiO_2_) NPs (NM101) were obtained from the nanomaterial repository of the European Commission’s Joint Research Centre (JRC). AgNO_3_ was purchased from Sigma (Sweden). Ag NPs (Sigma) and TiO_2_ NPs stock dispersions (1 mg/ml) were prepared in endotoxin-free water (Merck, Sweden) by probe sonication for 4 min whereas the other Ag NPs (NanoComposix) were dispersed directly in the desired medium without sonication. NPs were further diluted to achieve working concentrations in the respective media used for the various assays.

### 2.2 NP characterization

The morphology and crystallinity of Ag NPs was analyzed by transmission electron microscopy (TEM) (JEM-2100 F, 200 kV, JEOL, Tokyo, Japan). The samples were prepared by drop casting and drying 100 μl NPs on a copper grid as described (Saladino et al., 2021). Hydrodynamic size and surface charge (ζ-potential) of the various NPs was determined using the Zetasizer Nano ZS (Malvern, United Kingdom) in MilliQ^®^ water, Tris-HCl buffer (10 mM, pH 7.2), Dulbecco’s Modified Eagle Medium (DMEM), nasal epithelial cell medium (NECM), and PneumaCult™-Ex Plus Medium (see below for details). NP dispersions were prepared by diluting the stock solution to a working concentration (10 μg/ml) in the respective medium. The data presented are average values of three individual measurements. Fourier-transform infrared spectroscopy (FT-IR) measurements were performed using the Nicolet™ iS20 FTIR spectrometer (Thermo Scientific). Samples were prepared by mixing 3 mg of Ag NPs, or 1 ml of the peptide–Ag NP mixture, with 100 mg KBr and pressing into a pellet (KBr Mini-Pellet Press, Specac^®^, SigmaAldrich). The two 11-amino acid peptides corresponding to the S1/S2 junction of SARS-CoV (HTVSLLRSTSQ) and SARS-CoV-2 (TNSPRRARSVA), respectively, were purchased from GenScript (Leiden, Netherlands). FT-IR measurements were then performed on these pellets in the transmission mode, in the spectral range of 4,000 to 450 cm^−1^. The data were plotted using OriginPro^®^ software (OriginLab Corp. Northampton, MA, United States). Thermal gravimetric analysis (TGA) was performed on Ag NPs from Sigma from RT up to 670°C under inert atmosphere (N2 gas flow of 20 ml/min), using the TGA 550 system (TA Instruments, Sweden). Evolved gas analysis (EGA) was performed for the identification of functional groups using a TGA-IR coupling system (Thermo-Fisher) connected to a Nicolet™ iS10 FTIR spectrometer (Thermo Scientific).

### 2.3 Endotoxin assay

NPs were assessed for lipopolysaccharide (LPS) content using the chromogenic endpoint Limulus Amebocyte Lysate (LAL) assay (Lonza, Walkersville, MD, United States) (Bhattacharya et al., 2017). The absorbance was measured on a Tecan Infinite^®^ F200 spectrophotometer (Männedorf, Switzerland). The NPs were all endotoxin-free.

### 2.4 Virus neutralization assay

The pseudovirus neutralization assay was performed using ACE2-expressing HEK293T cells according to established protocols (Hanke et al., 2020). Briefly, pseudotyped viruses sufficient to produce ±100,000 RLU were incubated with serial 3-fold dilutions of Ag NPs and TiO_2_ NPs for 60 min at 37°C. Then, approximately 15,000 HEK293T-ACE2 cells were added to each well and the plates were incubated at 37°C for 48 h. The cells were maintained in DMEM supplemented with 10% fetal bovine serum (FBS) (Gibco). Alternatively, to identify effects mediated *via* target cells, cells were first incubated with pseudovirus for 24 h and thereafter the medium was replaced, and NPs were added. Luminescence was measured using the Bright-Glo™ Luciferase Assay System (Promega) with a Glo-Max^®^ luminescence plate reader (Promega). Inhibition was calculated relative to the average of control wells infected in the absence of serum. Spike-pseudotyped lentivirus particles were generated by the co-transfection of HEK293T cells with a relevant spike plasmid, an HIV gag-pol packaging plasmid (addgene #8455), and a lentiviral transfer plasmid encoding firefly luciferase (addgene #170674). For the lung cell assay, H1299 cells were stably transduced to express human ACE2 and TMPRSS2 (H1299-ACE2-TMPRRS2) by lentiviral transduction with pWPI-IRES-Puro-Ak-ACE2-TMPRSS2 (addgene plasmid #154987) and puromycin selection as described recently by us (Sheward et al., 2021). The cells were cultured in Dulbecco’s Modified Eagle Medium (DMEM) (containing high glucose, with sodium pyruvate) supplemented with 10% FBS, 100 U/mL penicillin, and 100 μg/ml streptomycin.

### 2.5 Recombinant spike (S) protein

Recombinant SARS-CoV-2 S-protein was synthesized and purified as described (Hanke et al., 2020). The plasmid for the expression of the S-protein encodes residues 1–1,208 of 2019-nCoV S (GenBank: MN908947) with proline substitutions at residues 986 and 987 resulting in a prefusion conformation (Wrapp et al., 2020). To assess interactions with various NPs, purified S-protein (20 μg/ml) was incubated with NPs (10 μg/ml) in 10 mM Tris-HCl buffer (pH 7.2) for 1 h and subjected to ζ-potential measurements and CD spectroscopy (below).

### 2.6 Circular dichroism (CD) spectroscopy

The secondary structure of the recombinant spike (S) protein was assessed by CD spectroscopy (Jasco, J-810 spectropolarimeter). To this end, the S-protein was incubated with and without Ag or TiO_2_ NPs (or AgNO_3_) in Tris-HCl buffer (10 mM, pH 7.2) and the CD measurements were performed at 25°C. Spectra were collected in the far-UV spectral region (190–250 nm). Each sample was measured six times, and averaged spectra are displayed.

### 2.7 Primary human nasal epithelial cells

Primary human nasal epithelial cells (HNEC) (cat no. C-12620) were obtained from Sigma (Sweden). These cells have been cryopreserved at cell passage 2 and tested for the absence of virus infections and microbial contaminants (fungi, bacteria, mycoplasma) prior to delivery. HNEC were maintained in airway epithelial cell growth medium (cat. no. C-21060) from Sigma (Sweden). The cell medium is also tested for the absence of microbial contaminants (fungi, bacteria, mycoplasma). The cell medium (referred to hereafter as NECM) is serum-free and contains the following supplements: bovine pituitary extract: 0.004 ml/ml; epidermal growth factor (recombinant, human): 10 ng/ml; insulin (recombinant, human): 5 μg/ml; hydrocortisone: 0.5 μg/ml; epinephrine: 0.5 μg/ml; triiodo-L-thyronine: 6.7 ng/ml; transferrin, holo (human): 10 μg/ml; retinoic acid: 0.1 ng/ml. The experiments were all performed using HNEC between passages 5 and 10.

### 2.8 Transformed bronchial epithelial cells

The immortalized human cell line BEAS-2B was obtained from European Collection of Cell Cultures. The cells were cultured in PneumaCult™-Ex Plus Medium (Stemcell Technologies, United Kingdom) supplied with 50x extra supplement; hydrocortisone (Stemcell Technologies), and penicillin-streptomycin (Gibco, Sweden) (Mukherjee et al., 2020). Cells were seeded at a density of 25,000 cells/cm2, and cytotoxicity was evaluated using the Alamar blue and LDH release assays, as described below.

### 2.9 Serum and lung surfactant corona

To evaluate potential protein corona formation, the NPs were incubated in 10% FBS in MilliQ^®^ water for 1 h at 37°C at 100 μg/ml. Following incubation, the unbound FBS was removed by centrifugation at 20,000 × g for 30 min, and the pellet was redispersed in MilliQ^®^ water by vortexing for 1 min. Next, measurements of hydrodynamic size and zeta potential were carried out as above. Samples for high-resolution (HR) TEM were also prepared as described above and the adsorbed layer of FBS on NPs was visualized using TEM (JEM-2100 F, 200 kV, JEOL). For pulmonary surfactant studies, CuroSurf^TM^ (80 mg/ml) was obtained from Chiesi Pharma GmbH (Hamburg, Germany). Ag NPs (1 mg/ml) were incubated overnight with CuroSurf^TM^ (1.2 mg/ml) at 37°C as described previously (Bhattacharya et al., 2015). Then, unbound CuroSurf^TM^ was removed by centrifugation at 20,000 × g for 30 min. CuroSurf^TM^-Ag NP complexes were redispersed in MilliQ^®^ water and washed once to remove any loosely bound biomolecules. Next, hydrodynamic size and zeta potential measurements were carried out as described above. The pseudovirus and cytotoxicity assays were performed with Ag NPs incubated or not with CuroSurf^TM^, as described in previous sections.

### 2.10 Western blotting

For protein detection, 25,000 cells/cm^2^ were seeded in a 6-well plate and exposed to NPs at the indicated concentrations for 24 h. Following exposure, cells were collected and lysed overnight at 4°C in RIPA buffer [50 mM Tris HCl (pH 7.4), 150 mM NaCl, 1% Triton X-100, 0.25% sodium deoxycholate, 0.1% SDS, 1 mM EDTA]. Protease- and phosphatase inhibitors (Mini EDTA-free Protease Inhibitor Cocktail, Sigma Aldrich; 1 mM PMSF, Thermo Fisher; PhosSTOP™, Sigma Aldrich) and 1 mM DTT (Sigma Aldrich) were freshly added to the buffer. Cell lysates were centrifuged at 13.000 × g for 15 min and supernatants were collected. The protein concentration was measured using the Bradford assay and 30 µg were loaded into each well of a NuPAGE 4%–12% Bis-Tris gradient gel (Thermo Fisher). Following electrophoretic separation, the proteins were transferred to a Hybond Low-fluorescent 0.2 µm PVDF membrane (Amersham), blocked for 1 h in Odyssey^®^ Blocking Buffer (PBS) (LI-COR), and stained overnight at 4°C with primary antibodies against NRF2 (Abcam, ab62352). Antibodies against β-actin (Sigma-Aldrich) were used for loading control, and the goat anti-mouse IRDye 680RD antibody (LI-COR Biotechnology GmbH, Bad Homburg, Germany) was used as a secondary antibody. Proteins were detected using LI-COR Odyssey^®^ CLx scanner and Odyssey^®^ Image Studio software.

### 2.11 Optical and fluorescence microscopy

For optical and fluorescence microscopy of HNEC, cells were seeded on glass coverslips placed in a 24-well plate. The cells were then exposed as indicated. After exposure, the cells washed with PBS and fixed with paraformaldehyde (4%). Then, the cells were washed and stained with phalloidin red (Abcam) for 15 min and counterstained and mounted with ProLong™ Gold Antifade Mountant containing DAPI (Thermo Fisher Scientific) and imaged using the EVOS™ M7000 imaging system (Thermo Fisher Scientific) at ×400 magnification.

### 2.12 Cell viability/metabolic capacity

For cytotoxicity assessment, cells were seeded in 96-well plates at a density of 1.5 × 10^4^ cells/cm^2^ 1 day before the experiment. The following day, NP dispersions were prepared in cell culture medium and added to the cells to achieve final concentrations of 0, 0.01, 0.1, 1, 5, 10, 20, and 50 μg/ml. Following 24 or 48 h of exposure, supernatants of exposed cells were collected in a fresh 96-well plate and lactate dehydrogenase (LDH) release was monitored (Mukherjee et al., 2020) using the CytoTox 96^®^ Non-Radioactive Cytotoxicity Assay kit (Promega). Samples were analyzed using a Tecan Infinite^®^ F200 spectrophotometer (Männedorf, Switzerland). Results are expressed as % LDH release versus maximum LDH release (cell lysis). The Alamar blue assay for metabolic capacity was also performed. Cells exposed as above were incubated with the Alamar blue dye (Thermo Fisher Scientific) for 4 h, and fluorescence was analyzed using a Tecan plate reader as described elsewhere (Keshavan et al., 2021).

### 2.13 Inductively coupled plasma mass spectrometry (ICP-MS)

Cellular and acellular measurement of Ag was performed by ICP-MS following protocols previously described (Gupta et al., 2022). In brief, HNEC were seeded in a 6-well plate at a density of 0.5 × 10^6^ cells per well 1 day before the experiment and then exposed to Ag NPs (10 μg/ml) and AgNO_3_ (eq. conc. of Ag 10 μg/ml) for 24 h. After exposure, cells were collected by trypsinization followed by washing three times with PBS and then processed for metal analysis by ICP-MS. For Ag ion release in water, NECM, and SNF, the NPs were dispersed at 10 μg/ml and incubated as indicated at 25°C. The simulated nasal fluid (SNF) contained NaCl 8.77 mg/ml, KCl 2.98 mg/ml, CaCl_2_ 0.59 mg/ml, pH 6.5. The samples were then centrifuged at 20,000 rpm for 1 h (4°C) and supernatants were carefully collected. Non-centrifuged dispersions were also collected and used as reference to determine the actual amount of Ag added. The samples were acid digested using HNO_3_ (32%) for 48 h to ensure complete mineralization. The final samples were diluted to achieve approximately 2% of HNO_3_. The Ag isotopes were quantified using an iCAP Q ICP-MS (ThermoScientific) instrument. Cell uptake results were normalized according to cell number and expressed as pg Ag/cell. Ag release was plotted as detected Ag in the respective medium in relation to the amount of Ag added.

### 2.14 Transmission electron microscopy

BEAS-2B cells exposed to NPs were processed for TEM as described (Gupta et al., 2022). Briefly, cells were seeded in 6-well plate 1 day before the experiment. The following day, cells were exposed to Ag-B NPs at 10 μg/ml for 24 h. Cells were collected and prefixed with 4% glutaraldehyde in 0.1 M sodium phosphate buffer pH 7.4 overnight at 4°C. Following post-fixation in 1% OsO_4_ in 0.1 sodium phosphate buffer for 2 h at 4°C, the cells were dehydrated using a gradient of ethanol followed by acetone and LX-112 infiltration and finally embedded in LX-112. Ultrathin sections were prepared using a Leica EM UC6 microtome, contrasted with uranyl acetate followed by lead citrate, and examined using a Hitachi HT 7700 electron microscope (Hitachi High-Technologies). Digital images were acquired using a 2kx2k Veleta charge-coupled device (CCD) camera (Olympus).

### 2.15 Statistics

The results shown are derived from at least three independent experiments. Data are presented as mean values ±S.D. GraphPad Prism 5 was used for the analysis. One-way ANOVA followed by Dunnett’s or Tukey’s *post hoc* analysis was applied.

## 3 Results and discussion

### 3.1 Ag NPs and their stability in relevant biological media

The bare Ag NPs (denoted as Ag-B NPs) were hetero-distributed in the range of 10–100 nm, in accordance with the information provided by the manufacturer (not shown). High-resolution TEM images displayed in Supplementary Figure S1A provided further insight into the morphology and crystallinity of the Ag NPs. The Ag-B NPs thus showed a high crystallinity as evidenced by the visible lattice fringes. Based on fast Fourier-transform (FFT) analysis of the regions denoted in Supplementary Figure S1A, the crystalline nature of the Ag-B NPs could be confirmed. To understand the behavior of Ag NPs in relevant exposure media, the hydrodynamic size, zeta potential, and stability (i.e., changes in size distribution over time), were evaluated. The hydrodynamic diameter of the Ag-B NPs in dH_2_O, Tris-HCl (used for CD measurements), DMEM (used for pseudovirus assays), and NECM (used for nasal epithelial cell culture) was in the range of 200–300 nm immediately after dispersion; however, the size was slightly reduced at 24 h, possibly because of sedimentation of larger agglomerates formed in the different media (Supplementary Table S1). The Ag-B NPs displayed a negative surface charge in dH_2_O, Tris-HCl, and DMEM/NECM, with ζ-potential values of approx. −40, −30, and −10 mV, respectively (Supplementary Table S1). TiO_2_ NPs (NM101) from the nanomaterial repository at JRC were used as a control in some experiments, as described below, and the average hydrodynamic size of the latter NPs in the various exposure media was 400–850 nm (Supplementary Table S1). This was further increased at 24 h especially in Tris-HCl (used for CD spectroscopy), indicating that the particles agglomerated (Supplementary Table S1). The latter result is in agreement with previous work showing that TiO_2_ NPs aggregated more readily in the presence of anions than cations (Shih et al., 2012). TiO_2_ NPs displayed a positive ζ-potential in dH_2_O and a negative charge in DMEM/NECM comparable to the Ag NPs (Supplementary Table S1). This may be explained by the adsorption of the proteins in the medium (serum/FBS in DMEM, growth factors in NECM).

Next, we studied acellular dissolution of the Ag-B NPs in dH_2_O, NECM and simulated nasal fluid (SNF) using ICP-MS. We observed some dissolution of the Ag-B NPs (approx. 15%) in both NECM and SNF after 24 h (Supplementary Figure S1B). The dissolution of Ag NPs is known to be size-dependent in that 10 nm Ag NPs were shown in a previous study from our laboratory to release significantly more Ag as compared with 50 or 75 nm Ag NPs (approx. 25% versus 5%) after immersing the NPs for 24 h in serum-free bronchial epithelial cell growth medium (Gliga et al., 2014). Similarly, other investigators have shown that the release of Ag is directly related to the total surface of the particles and the composition of the exposure media (Liu et al., 2010; Liu et al., 2012). These findings are relevant as one considers the potential impact of Ag NPs on SARS-CoV-2 under *in vitro* conditions, or in a (future) clinical setting in human subjects. Indeed, a recent study using immunodeficient mice showed that dissolution of intranasally instilled Ag NPs affected their biodistribution *in vivo* (Zamborlin et al., 2022).

### 3.2 Ag NPs neutralize viruses pseudotyped with the S-protein

**FIGURE 1:**
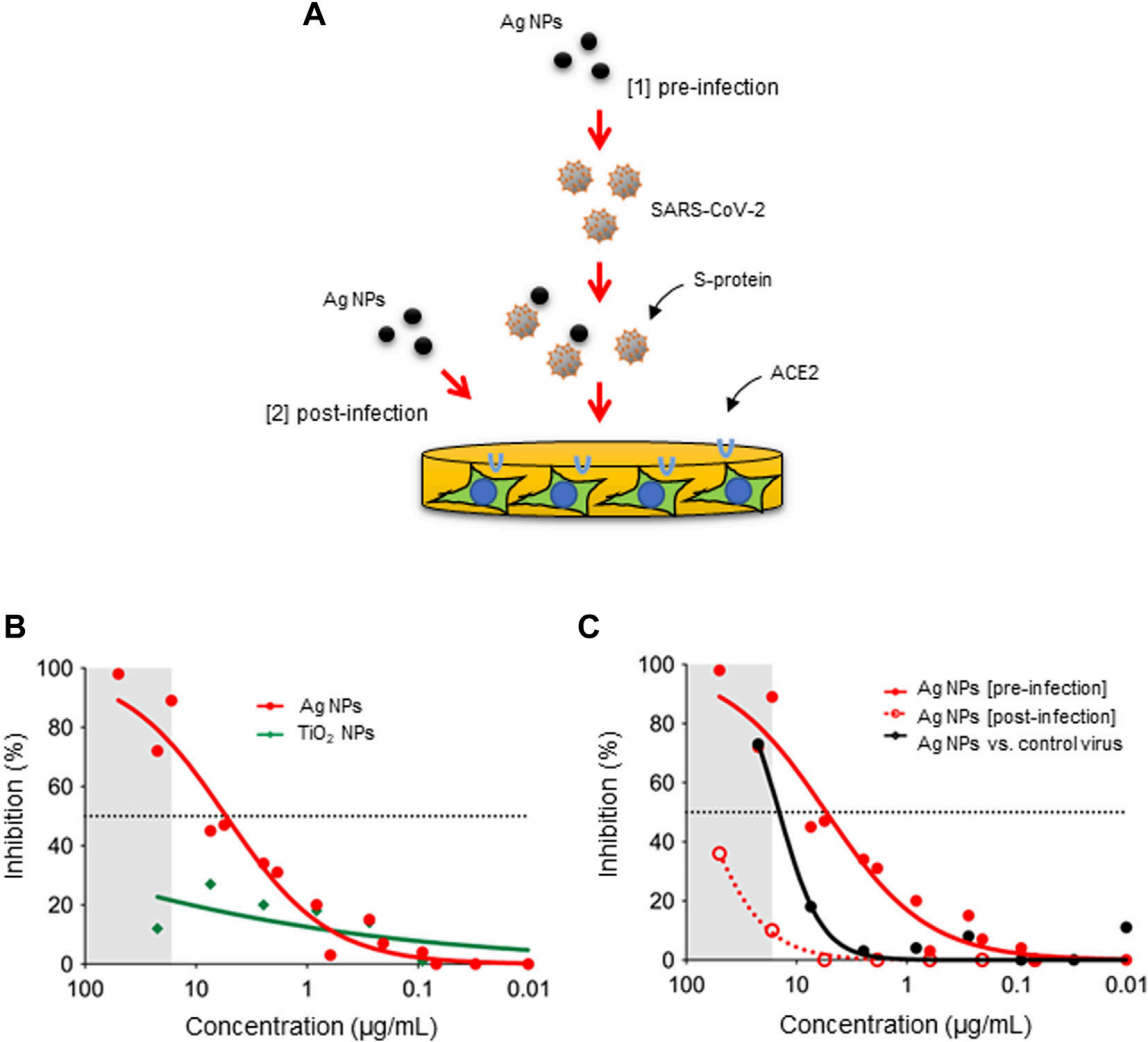
Ag NPs neutralize viruses pseudotyped with the S-protein of SARS-CoV-2. **(A)** The schematic figure shows the two different approaches applied in the present study, i.e., pre-incubation of the virus with the NPs (pre-infection) and addition of the NPs to the cells after the cells have been infected (post-infection). The pseudovirus neutralization assay was performed using ACE2-expressing HEK293T cells. **(B)** Inhibition of pseudovirus infection after pre-incubation with Ag-B NPs versus TiO_2_ NPs for 1 h. **(C)** Comparison of inhibition potential of Ag-B NPs under pre-infection and post-infection conditions. The dotted line represents the EC_50_ value (8.0 μg/ml). Additionally, Ag-B NPs were pre-incubated with the non-pseudotyped (control) virus. The grey area denotes concentrations at which reduced cell numbers were seen under the light microscope following exposure to pseudotyped virus pre-incubated with Ag-B NPs.

Metallic NPs including Ag NPs as well as functionalized gold (Au) NPs have been shown to exert anti-viral activities (Baram-Pinto et al., 2010; Lv et al., 2014; Vonnemann et al., 2014; Yang et al., 2016). Jeremiah et al. (2020) reported recently that Ag NPs prevented SARS-CoV-2 infection and suggested that the particles acted as viral entry inhibitors. Similarly, previous studies have shown that Ag NPs exert anti-HIV (human immunodeficiency virus) activity at an early stage of viral replication, while silver salts (i.e., AgNO_3_) were less efficient (Lara et al., 2010). Furthermore, it was suggested that Ag NPs were preferentially bound to the gp120 subunit of the viral envelope glycoprotein of HIV-1, though the basis for this interaction was not disclosed (Elechiguerra et al., 2005). Here we asked if Ag NPs can prevent SARS-CoV-2 infection using a pseudovirus neutralization assay (Figure 1A). To this end, we used lentiviral particles pseudotyped with the S-protein of SARS-CoV-2 (Hanke et al., 2020). As shown in Figure 1B, potent neutralization of SARS-CoV-2 pseudotyped viruses was observed when the Ag-B NPs had interacted with pseudotyped virus for 1 h (in DMEM) prior to infecting the HEK293T-ACE2 cells (EC50 ∼8.0 μg/ml). However, TiO_2_ NPs were found to be ineffective in preventing infection (Figure 1B). The latter result demonstrates that virus neutralization is not a general particle effect. Moreover, we found that Ag-B NPs were ineffective in preventing virus infection using the non-pseudotyped control virus, except at the highest concentration of the NPs (Figure 1C), indicating that the effect was specific for SARS-CoV-2. We noted that the combination of Ag NPs plus virus resulted in reduced cell numbers at high concentrations of the NPs (indicated as a grey zone in Figures 1B, C). Indeed, as the readout for infectivity relies on luciferase expression by target cells, compounds that are cytotoxic or otherwise inhibit gene expression can appear inhibitory in such assays. To address this, we exposed cells with Ag NPs 24 h after infection (Figure 1A). As shown in Figure 1C, the reduction in luciferase expression was markedly absent when the Ag-B NPs were added post-infection indicating that the inhibition of virus infection was not due to effects on target cells. Furthermore, this supports the notion that inhibition occurs at an early step, perhaps *via* direct interactions between the NPs and the virus. However, this does not preclude the possibility of cellular effects of Ag NPs. In fact, we recently demonstrated that small (10 nm) citrate-coated Ag NPs as well as AgNO_3_ inhibited bacterial lipopolysaccharide (LPS)-triggered Toll-like receptor signaling in human monocytes (Gliga et al., 2020). Notwithstanding, the effect of Ag-B NPs on virus infection was evident at an early (pre-infection) step in our pseudovirus neutralization assay, and at non-cytotoxic concentrations of the NPs.

**FIGURE 2:**
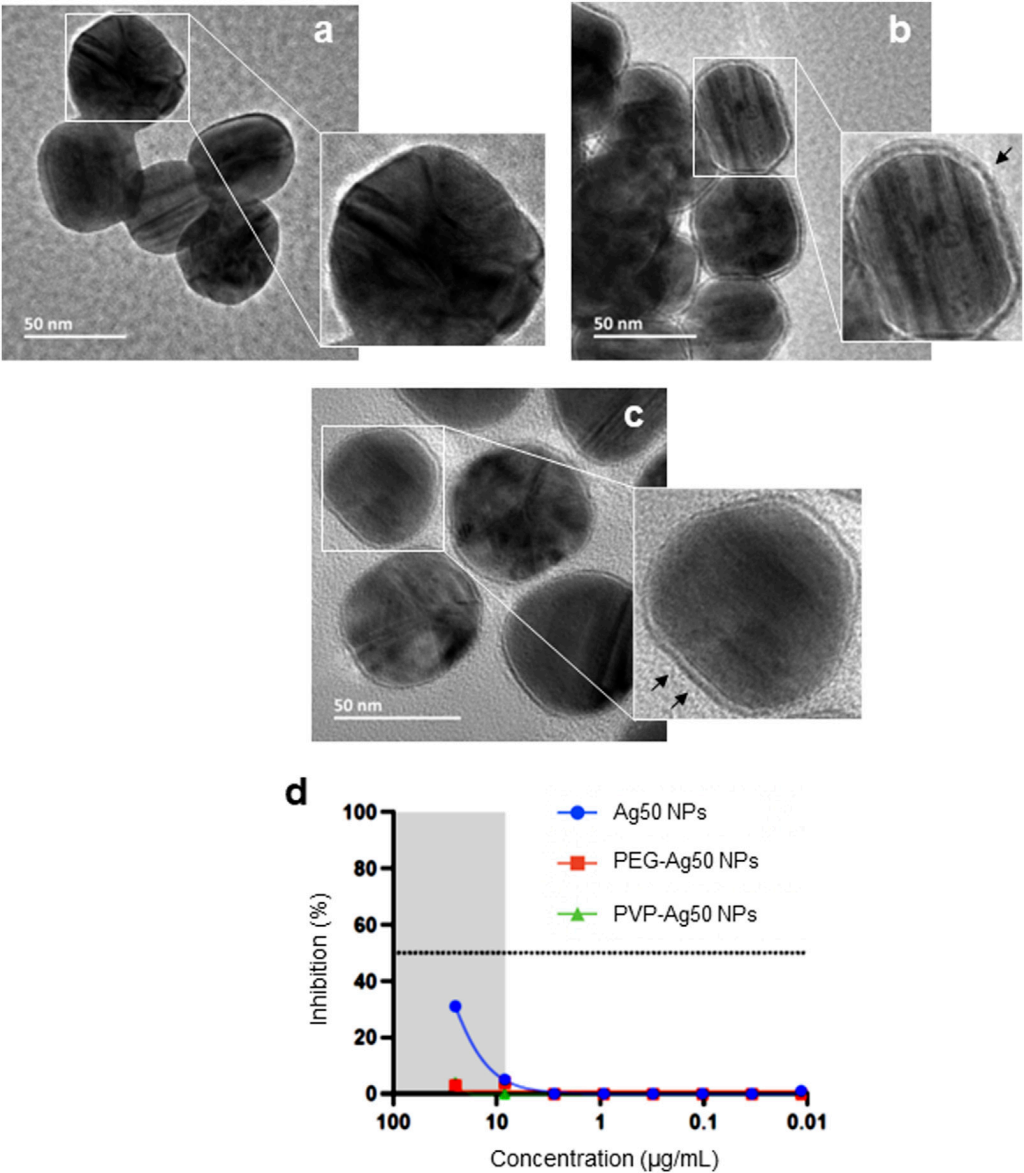
Surface coating impacts on anti-viral activity of Ag NPs. HR-TEM micrographs of **(A)** bare-Ag50 NPs (citrate-stabilized), **(B)** PVP-coated Ag50 NPs, and **(C)** PEG-coated Ag50 NPs. The black arrows in **(B,C)** denote the surface coating. **(D)** Inhibition of pseudovirus infection in the human lung cell model H1299-ACE2-TMPRSS2 cells after pre-incubation (1 h) with bare versus PVP- or PEG-coated Ag NPs.

We assumed that these effects were due to a direct interaction of the Ag NPs with the virus. To investigate the role of the particle surface, we used a set of Ag NPs of similar primary particle size (50 nm) and varying surface properties (i.e., unmodified, PVP-coated, or PEG-modified). These NPs (obtained from a different supplier as compared to the Ag-B NPs) were first characterized with respect to hydrodynamic diameter and surface charge in various media (dH_2_O, DMEM, and PneumaCult™) (Supplementary Figure S2). These studies showed that the Ag NPs were well dispersed and did not agglomerate in DMEM supplemented with 10% fetal bovine serum (FBS) (i.e., the cell medium that is used for the pseudovirus neutralization assay), while the unmodified and PVP-coated particles agglomerated in the PneumaCult™ medium that is used for the BEAS-2B lung cell line (see below). However, the PEGylated Ag nanoparticles did not display agglomeration in DMEM or PneumaCult™ (Supplementary Figure S2). The Ag NPs were imaged using HR-TEM and the surface coatings could be clearly visualized (Figures 2A–C). We then asked whether these NPs would neutralize the virus. To this end, the recently developed lung cell model, H1299-ACE2-TMPRRS2 (Sheward et al., 2021), was used instead of the HEK293T-ACE2 cell line. Notably, only the unmodified Ag NPs prevented infection in the pseudovirus neutralization assay while the PVP-coated and PEGylated Ag NPs were ineffective (Figure 2D). For comparison, a previous study (using NPs obtained from the same source, NanoComposix) has shown that Ag NPs coated with PVP or with branched polyethylenimine (PEI) were more virucidal than Ag NPs coated with citrate (He et al., 2022). Surface properties of Ag NPs can modulate the bio-corona (Barbalinardo et al., 2021). However, the potential impact of the bio-corona of proteins or other biomolecules on the anti-viral effects of the nanoparticles has not been evaluated previously.

### 3.3 Lung surfactant does not prevent the anti-viral activity

**FIGURE 3:**
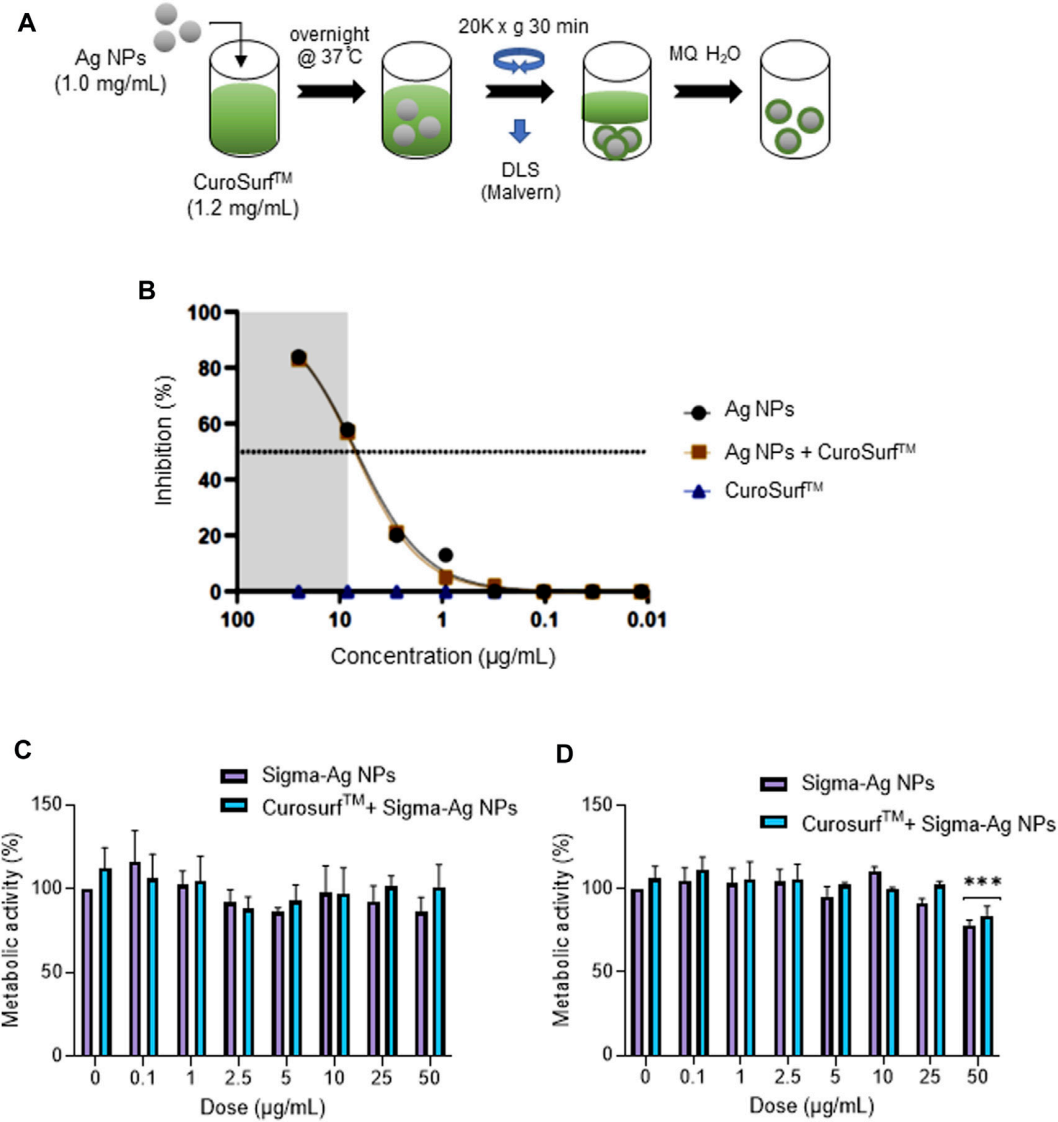
Lung surfactant does not interfere with the anti-viral activity of Ag NPs. **(A)** Schematic representation of the steps involved in formation of a lung surfactant bio-corona on Ag NPs. The CuroSurf™-Ag NPs complexes were analyzed with respect to hydrodynamic diameter and ζ-potential at each washing step (data not shown). **(B)** Ag-B NPs alone or with a lung surfactant bio-corona showed similar potency in the pseudovirus neutralization assay using the H1299-ACE2-TMPRSS2 model. Cytotoxicity (loss of metabolic activity) of Ag-B NPs with or without a bio-corona was evaluated in the BEAS-2B lung cell line at 24 h **(C)**, and 48 h **(D)**, using the Alamar blue assay.

The pseudovirus assays referenced above were performed using cells incubated in “standard” cell culture medium supplemented with 10% FBS, and it is well known that serum proteins are adsorbed onto the surface of NPs leading to a so-called bio-corona (Hansen and Thünemann, 2015; Sasidharan et al., 2015). However, in a real-life scenario, if and when particles are inhaled, they will encounter a different biological medium, leading to a bio-corona composed of pulmonary surfactant. To add realism to our *in vitro* model, we established a protocol for the incubation of Ag-B NPs in lung surfactant (Figure 3A). To this end, we used CuroSurf^TM^, an extract of natural porcine lung surfactant consisting of 99% polar lipids (mainly phospholipids) and 1% hydrophobic low molecular weight surfactant proteins. The samples were monitored by DLS at each washing step, and we noted that the hydrodynamic diameter of the NPs was slightly decreased upon incubation with CuroSurf^TM^, suggesting that the particles were deagglomerated, while the ζ-potential was slightly decreased, though the ζ-potential remained negative, indicating that biomolecules present in lung surfactant were adsorbed to the NPs (not shown). Using the H1299-ACE2-TMPRRS2 model, we then determined whether the presence of a bio-corona of lung surfactant would affect the anti-viral activity of the Ag-B NPs. We found that the Ag-B NPs were as effective with and without a lung surfactant corona (Figure 3B). Thus, while several studies have reported a “corona” effect on the cellular uptake of NPs (Barbalinardo et al., 2021; Zhao et al., 2021), the corona may not cover the particle surface completely. Indeed, it was previously shown, using polystyrene NPs, that the protein corona is not a dense shell covering the NPs but a loose network of proteins (Kokkinopoulou et al., 2017). Hence, it is possible that interactions between the Ag NP surface and the virus could still occur. We also monitored the biocompatibility of Ag NPs with or without a corona of lung surfactant using the human immortalized bronchial epithelial cell line BEAS-2B. The NPs did not affect cell viability at 24 h (Figure 3C) while at 48 h, there was a drop in cell viability at the highest dose (50 μg/ml) for NPs with or without a pre-adsorbed bio-corona (Figure 3D).

**FIGURE 4:**
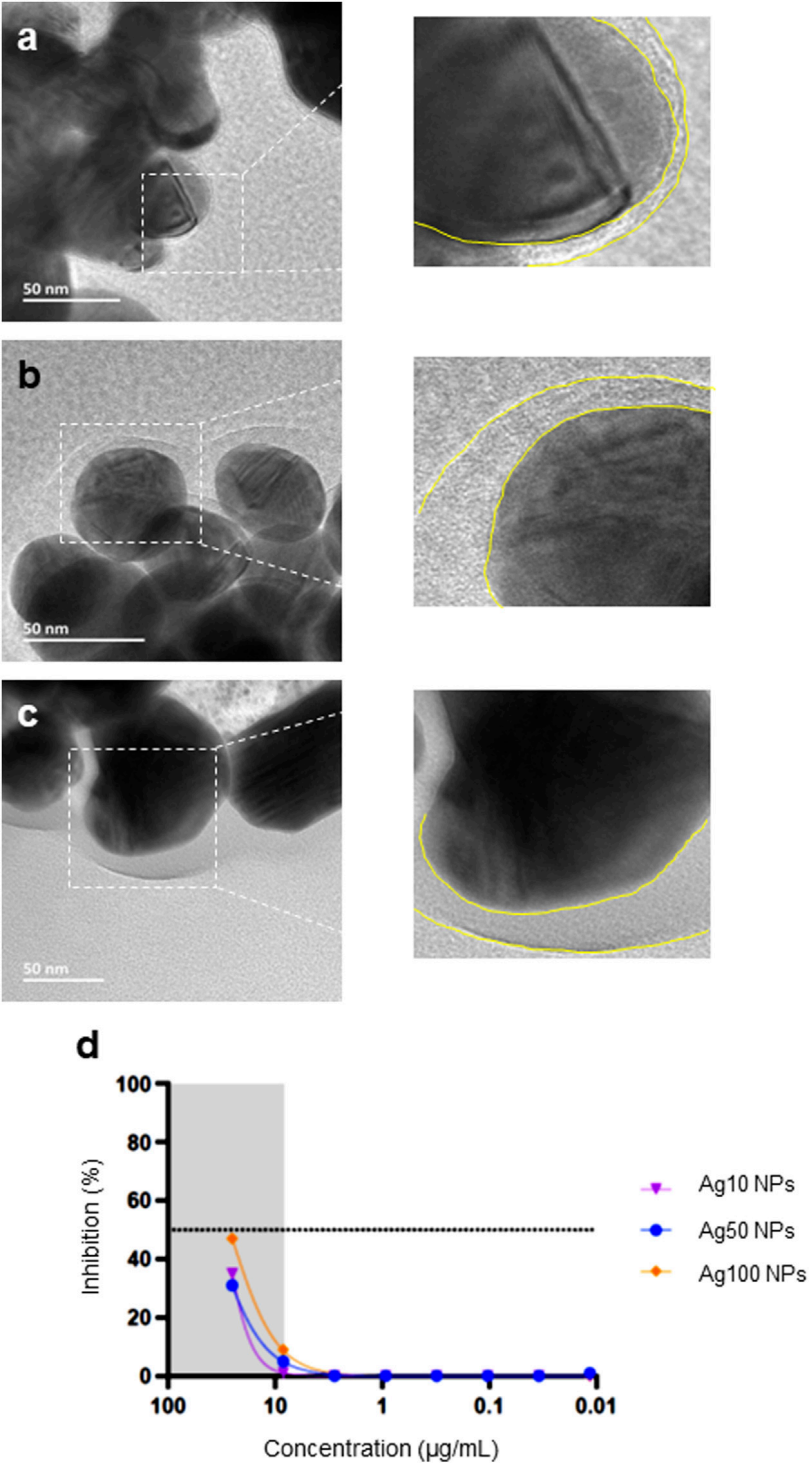
Bio-corona formation on Ag NPs. Ag NPs were incubated in 10% FBS in MilliQ^®^ water for 1 h at 37°C at 100 μg/ml and FBS-Ag NP complexes were isolated and washed once by centrifugation. The bio-corona on the Ag NPs surfaces was visualized by HR-TEM: **(A)** Ag-B (Sigma), **(B)** Ag50 (NanoComposix), and **(C)** Ag100 (NanoComposix). Note that all the Ag NPs tested were bare (uncoated) (see main text). The yellow dotted lines in the magnified images serve to show the thickness of the bio-corona. **(D)** Pseudovirus neutralization assay using bare Ag NPs of varying particle diameters (NanoComposix). The virus was pre-incubated with the Ag NPs for 1 h.

To further understand whether the Ag NPs from the two different commercial sources behaved differently in contact with biologically relevant media, we addressed the bio-corona formation that is known to occur when NPs are immersed in cell culture medium. NPs were thus incubated with FBS in MilliQ^®^ water and visualized using HR-TEM. The surface-adsorbed layer of organic matter could be clearly seen (Figures 4A–C). Moreover, the thickness of the adsorbed protein layer was greater for the citrate-stabilized 50 and 100 nm Ag NPs from NanoComposix when compared to the Ag-B NPs from Sigma. In addition, we evaluated a set of unmodified Ag NPs of different primary particle diameters (10, 50, and 100 nm) from NanoComposix using the H1299-ACE2-TMPRRS2 assay and found that the NPs were similar in terms of anti-viral activity, with the larger NPs being slightly more effective (Figure 4D). However, none of these NPs were as effective as the Ag-B NPs from Sigma (see above).

Our HR-TEM images of the Ag-B NPs suggested the possible presence of a thin, organic layer on the surface of the NPs obtained from Sigma. To begin to address this, we performed TGA and observed a total weight loss of about 4.5%, which might be due to an organic capping agent (Supplementary Figure S3A). To identify the putative degradation products, we performed evolved-gas analysis (EGA) by coupling the outlet of the TGA system with FTIR analysis (Supplementary Figure S3B). We selected a representative point in time to identify typical gaseous products and the corresponding spectrum is shown in Supplementary Figure S3C. The observed bands can be attributed to the presence of C-H, CO2, C=O and ether groups, which are typical degradation products of carbohydrates. Taken together, we cannot exclude the presence of an organic surface layer on the “bare” Ag NPs.

### 3.4 Ag NPs alter the secondary structure of the S-protein

Cell entry of coronaviruses depends on binding of the S-protein complexes that form the eponymous “crown” to the corresponding cellular receptors and on S-protein priming by host cell proteases (Du et al., 2009). Specifically, the S-protein of SARS-CoV-2 binds to ACE2 and is processed by cellular proteases including TMPRSS2 (transmembrane protease, serine 2) (Hoffmann et al., 2020a) and furin (also known as “paired basic amino acid cleaving enzyme” or PACE) (Hoffmann et al., 2020b; Shang et al., 2020). The conformation of the S-protein is thus important for the infectivity of SARS-CoV-2. Ag NPs have previously been shown to trigger conformational changes in proteins. For instance, small (5–10 nm) Ag NPs were found to cause conformational changes of hemoglobin, as evidenced by CD spectroscopy (Devi et al., 2014). Moreover, using synchrotron radiation circular dichroism, Laera et al. (2011) could show that human serum albumin is destabilized when interacting with Ag NPs, while its stability was not affected by Au NPs. To investigate whether the Ag NPs triggered any changes in the secondary structure of the S-protein of SARS-CoV-2, we performed CD spectroscopy of recombinant S-protein incubated with Ag-B NPs in Tris-HCl (pH 7.2). The S-protein forms an α-helical coiled-coil secondary structure at the C-terminal region. We found that the Ag-B NPs triggered a marked decrease in the α-helical content (208 nm) of the S-protein (Supplementary Figure S4A), and an increase in the random coil (195 nm) content (Supplementary Figure S4B). The appearance of random coils is indicative of denatured proteins. TiO_2_ NPs did not trigger such changes in the S-protein (Supplementary Figures S4C, D), and the equivalent concentration of AgNO_3_ was found to be ineffective (Supplementary Figures S4E, F), suggesting that the effects observed were not due to a general particle effect or caused by Ag ions. Neither the NPs nor the soluble salt interfered with the CD measurements (Supplementary Figures S5A–F).

Silver staining is a classical method for the detection of proteins separated on gels and membranes (Merril, 1990), and the interaction of “bare” or unmodified Ag NPs with proteins is not unexpected. However, while cysteine and methionine are commonly believed to be the preferential binding sites for Ag ions in proteins, theoretical and experimental studies have shown that the three basic amino acids arginine, lysine and histidine are, in fact, the strongest Ag binders (Shoeib et al., 2002). It is worth noting that the S-protein contains a so-called multi-basic (arginine-rich) motif that serves as a cleavage site for furin as well as other cellular proteases (Jaimes et al., 2020). Furthermore, it was recently shown that loss of this cleavage site attenuates the pathogenicity of SARS-CoV-2 (Johnson et al., 2021). To test whether the Ag-B NPs could interact with this arginine-rich motif, we prepared peptides corresponding to the S1/S2 junction of SARS-CoV and SARS-CoV-2, respectively (Supplementary Figure S6A). Aliquots of Ag-B NPs were then added to these peptide solutions and analyzed using solid-state FT-IR spectroscopy. The 1800–1,500 cm^−1^ region corresponds to the stretching band of amide I (C=O stretching; 1,600–1,800 cm^−1^) and the bending peak of amide II (C–N stretching and N–H bending; 1,500–1,600 cm^−1^) whereas the 3,500–3,200 cm^−1^ region is important for assessing N–H stretching vibrations, which provides hydrogen bonding information in peptides and proteins. The spectra for the amide I and amide II regions for samples containing the SARS-CoV peptide plus Ag-B NPs (Supplementary Figures S6B, D) and the SARS-CoV-2 peptide plus Ag-B NPs (Supplementary Figures S6C, E) are presented. FT-IR spectra for the NPs alone are shown in Supplementary Figures S7A, B. First, an important common feature is observed in the 1,600–1800 cm^−1^ region (C=O stretching), with a dramatic reduction of the absorption band at 1,620 cm^−1^ in the presence of the Ag-B NPs. This effect was more pronounced in the case of the SARS-CoV-2 peptide as compared to the SARS-CoV peptide, as can be seen from the amplitude ratio of the observed bands at 1,620 and 1,660 cm^−1^ (Supplementary Figures S6B, C). This effect was also seen on the N–H bands, with a significant reduction of the absorption band at 3,300 cm^−1^ which is ascribed to the intramolecularly hydrogen bonded N–H stretching vibrations in the case of the SARS-CoV-2 peptide (Supplementary Figures S6D, E). This reduction may be attributed to the shielding of H bonds which play a significant role in the formation of secondary structures. It is conceivable that similar interactions with the multi-basic motif at the S1/S2 junction of the S-protein could explain the observed inhibition of virus entry. However, this domain is not necessarily the only point of interaction between the Ag NPs and the virus. The unfolding of the S-protein that we documented here could serve to mask the multi-basic motif thereby preventing infection of host cells. The interactions between Ag NPs and proteins are complex and NP properties (size, shape, surface modifications) as well as the properties of the proteins may come into play (Barbir et al., 2021). Further studies are required to pin down the molecular mechanism of the observed anti-viral effects of Ag NPs.

### 3.5 Ag NPs are non-cytotoxic for human epithelial cells

**FIGURE 5:**
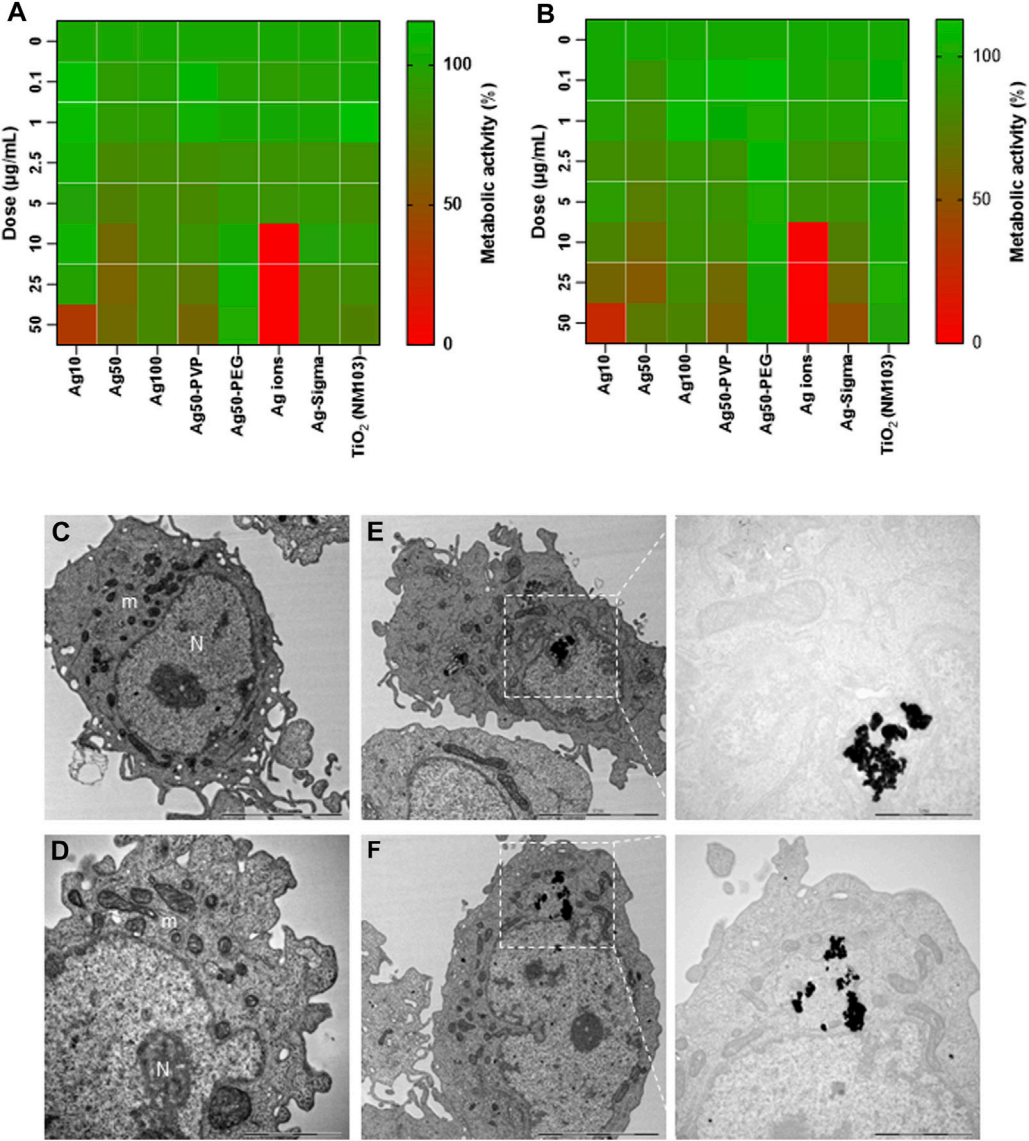
Biocompatibility assessment of a panel of NPs. Cytotoxicity of Ag NPs of different sizes and varying surface modifications (as indicated) was evaluated using the human bronchial epithelial cell line BEAS-2B. TiO_2_ NPs and AgNO_3_ were included as controls. Cytotoxicity (loss of metabolic capacity) was determined by Alamar blue assay after 24 h **(A)** and 48 h **(B)** of exposure of the cells. The results shown are mean values of three independent experiments, plotted as heatmaps. **(C–F)** TEM imaging. BEAS-2B cells were maintained in medium alone **(C,D)** or were exposed to Ag-B NPs (10 μg/ml) for 24 h **(E,F)**. Electron-dense clusters of Ag NPs are clearly seen in **(E,F)**. N, nucleus; m, mitochondria. Scale bars: 5 µm **(C,E,F)**; 2 µm [**(D)**, magnified portion of panel **(F)**]; 1 µm [magnified portion of **(E)**].

**FIGURE 6:**
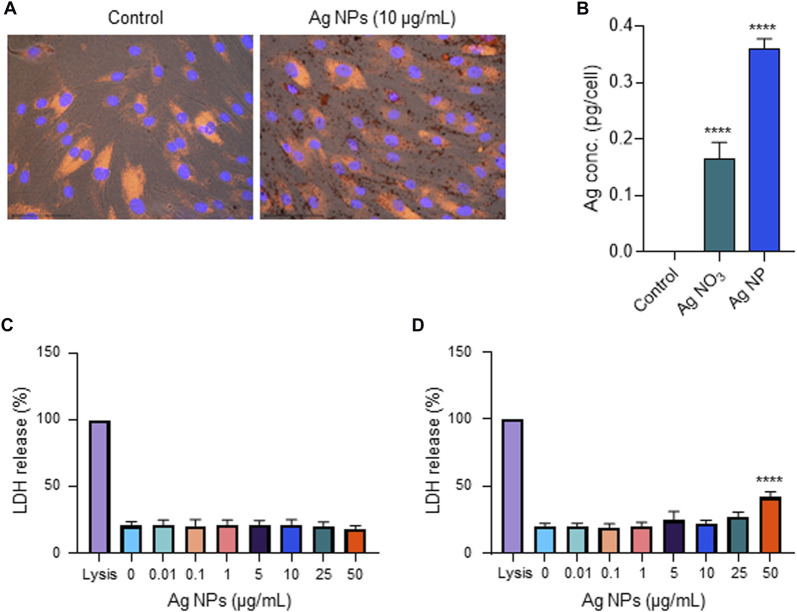
Biocompatibility assessment using primary cells. **(A)** Cellular uptake of Ag-B NPs (25 μg/ml) in primary human nasal epithelial cells (HNEC) was determined at 24 h using fluorescence microscopy. DAPI (blue): cell nuclei; phalloidin red: actin. The dark granules in the panel to the right are suggestive of cellular uptake and/or cellular attachment of the Ag NPs. **(B)** ICP-MS showed increased intracellular Ag content in cells exposed to Ag-B NPs (10 μg/ml), and a lower cellular content in cells exposed to AgNO_3_ at the equivalent concentration. Data presented are mean values ±S.D. (*n* = 3). ****p < 0.0001. **(C,D)** Cell viability of HNEC maintained in serum-free NECM was evaluated using the LDH release assay following exposure to Ag-B NPs for 24 h **(C)** and 48 h **(D)**, respectively. Data shown are mean values ±S.D. (*n* = 3). ****p < 0.0001.

We and others have previously shown that Ag NPs may elicit cytotoxicity in mammalian cells. However, these effects were size-dependent. Thus, we previously observed that 10 nm but not 50 or 75 nm Ag NPs triggered a loss of cell viability in human lung epithelial cells (Gliga et al., 2014). However, the same NPs were shown to be non-cytotoxic for mouse embryonic stem cells (Karlsson et al., 2014). Similarly, others have reported, using a panel of Ag NPs ranging from 10 to 100 nm, that genotoxicity inversely correlated with particle size (Butler et al., 2015). Furthermore, surface coating of the NPs also matters as this may affect dissolution (Wang et al., 2014). To evaluate the biocompatibility of the tested NPs, we screened all the NPs including the Ag-B NPs from Sigma and the TiO_2_ NPs along with the Ag NPs of varying sizes and surface modifications from NanoComposix using the BEAS-2B cell line. Cell viability was monitored at 24 h (Figure 5A) and 48 h (Figure 5B) using the Alamar blue assay. The Ag-B NPs from Sigma and the TiO_2_ NPs were not cytotoxic at 24 or 48 h in this cell model. For the NPs from NanoComposix, we found a size-dependent cytotoxicity, in line with previous reports (Gliga et al., 2014). It is also noted that PEGylation mitigated the cytotoxicity of the Ag NPs towards BEAS-2B cells. However, the Ag salt was by far the most cytotoxic of the tested materials (Figures 5A, B). TEM analysis of BEAS-2B cells exposed for 24 h to the Ag-B NPs (10 μg/ml) revealed the uptake of NPs without any ultrastructural signs of cell death (Figures 5C, D: control cells; Figures 5E, F: cells exposed to Ag-B NPs). To evaluate whether the Ag NPs could trigger protective cellular responses in these cells, we investigated whether the Ag-B NPs obtained from Sigma triggered an antioxidant response in the BEAS-2B cell line. As shown in Supplementary Figure S8, a clear dose-dependent upregulation of NRF2 was observed at 24 h, in line with other recent studies using different cell lines (Miranda et al., 2022). However, no induction was seen in cells exposed to the TiO_2_ NPs. This is of interest as a recent report has shown that the NRF2 antioxidant gene expression pathway is suppressed in the lungs of COVID-19 patients (Olagnier et al., 2020). Furthermore, the authors could show that NRF2 agonists including dimethyl fumarate (DMF) induced a cellular anti-viral program that potently inhibited replication of SARS-CoV-2. It can thus be speculated that Ag NPs act as NRF2 agonists, although our pseudovirus assay results showed that the anti-viral effect was due mainly to the prevention of virus infection of host cells. To further corroborate the biocompatibility of the Ag-B NPs from Sigma, we used primary human nasal epithelial cells (HNEC). These cells were selected because ACE2 and TMPRSS2 are both highly expressed in nasal epithelial cells (Sungnak et al., 2020). In fact, Hou et al. (2020) were able to show the highest ACE2 expression in the nose with decreasing expression throughout the respiratory tract. Hence, the nasal epithelium represents a key target for SARS-CoV-2. First, we studied cellular uptake of the NPs. Based on our microscopic analysis, the NPs appeared to be internalized (or attached) following 24 h of exposure (Figure 6A). To verify uptake, we performed ICP-MS. As shown in Figure 6B, an increase in intracellular Ag was observed when cells were exposed to Ag NPs (10 μg/ml). The cellular content was lower for AgNO_3_ (added at an equivalent concentration), supporting the view that the NPs are more readily internalized than Ag ions (Gliga et al., 2014). Next, we investigated the impact of the Ag-B NPs at 24 h (Figure 6C) and 48 h (Figure 6D) using the LDH release assay and found that the NPs were non-cytotoxic at 24 h. We noted a modest loss of cell viability at 48 h when cells were exposed at the highest concentration (50 μg/ml). We were not able to evaluate the Ag salt using the LDH release assay due to assay interference. Instead, we applied the Alamar blue assay, and we confirmed that the Ag-B NPs were non-cytotoxic towards HNEC (at 24 h) at concentrations ranging from 0.01 to 50 μg/ml (Supplementary Figure S9A) while AgNO_3_ triggered a pronounced dose-dependent reduction of cell viability (Supplementary Figure S9B). We also tested the TiO_2_ NPs using the Alamar blue and LDH release assays and found no cytotoxicity at 24 h (Supplementary Figures S10A, B), while a very modest response was observed at 48 h (Supplementary Figure S10C, D). Taken together, we have shown, using an immortalized human bronchial epithelial cell line (BEAS-2B) as well as primary human nasal epithelial cells, that Ag-B NPs (which displayed limited dissolution) are non-cytotoxic at the concentrations used to inhibit infection using pseudotyped viruses, while the Ag salt triggered dose-dependent toxicity (from 10 μg/ml).

There are some limitations to the present study. First, the fact that the Ag NPs were obtained from different commercial sources, making it difficult to compare the results of the pseudovirus assays. However, the comparison between the various NPs from NanoComposix remains valid, and the comparison between the “bare” Ag NPs from Sigma with and without lung surfactant, and the soluble Ag salt, also remains relevant. The chemical nature of the surface of the latter, so-called bare Ag NPs could not be discerned; this is a common concern when working with nanomaterials obtained from commercial sources (Allen et al., 2010). Nevertheless, the Ag-B NPs were found to effectively prevent virus infection, while sparing host cells (bronchial epithelial cells, and nasal epithelial cells). Furthermore, while we have shown that viruses pseudotyped with the S-protein are blocked by the Ag-B NPs, and while the same NPs were also shown to interact with peptides corresponding to the muti-basic motif, we cannot draw definitive conclusions regarding whether the Ag-B NPs interacted with a particular domain of the S-protein. However, our studies using surface-modified (PVP- or PEG-coated) Ag NPs (obtained from a different source) showed that interactions between the S-protein and the particle surface are important.

## 4 Conclusion

Our results demonstrated a potent anti-SARS-CoV-2 activity of Ag NPs as evidenced using two different pseudovirus neutralization assays. This activity could not be explained by soluble Ag ions as the Ag NPs were found to undergo limited dissolution. Moreover, as a first step towards uncovering the mechanism, we could show that Ag NPs but not AgNO_3_ perturbed the secondary structure of the S-protein which could interfere with proteolytic processing of the S-protein that is required for infection of host cells (Hoffmann et al., 2020a; Hoffmann et al., 2020b), and/or with the binding of cellular receptors such as ACE2. Furthermore, we could show that the anti-viral activity of the Ag NPs remained intact in the presence of pulmonary surfactant. On the other hand, PVP- or PEG-modified Ag NPs, failed to block virus infection in the present model, though it is notable that a recent study in which health workers were instructed to perform a mouth and nasal wash using PVP-coated Ag NPs suggested a protective effect against SARS-CoV-2 infection (Almanza-Reyes et al., 2021). We could also show, using primary human nasal epithelial cells, that the Ag NPs (unlike AgNO_3_) were non-cytotoxic at 24 h and displayed very modest toxicity at 48 h at the highest concentration, but not at the concentrations used to inhibit virus infection. Taken together, these findings have implications for the development of nano-enabled strategies to limit the spread of SARS-CoV-2 (Padín-González et al., 2022).

## Data Availability

The original contributions presented in the study are included in the article/Supplementary Material, further inquiries can be directed to the corresponding author.
