# Supplementary material for: Silver nanoparticles with excellent biocompatibility block pseudotyped SARS-CoV-2 in the presence of lung surfactant
Source: Front Bioeng Biotechnol. 2022 Dec 12;10:1083232. doi: 10.3389/fbioe.2022.1083232 (PMC9790969; doi:10.3389/fbioe.2022.1083232)
Supplement: Supplementary file 1 [file DataSheet1.PDF]

Supporting Information

Silver nanoparticles with excellent biocompatibility block pseudotyped SARS-CoV-2 in the presence of lung surfactant

Govind Gupta, Bejan Hamawandi, Daniel J. Sheward, Benjamin Murrell, Leo Hanke, Gerald McInerney, Magda Blosi, Anna L. Costa, Muhammet S. Toprak, Bengt Fadeel

Contents:

Hydrodynamic size and ζ-potential of Ag-B NPs and TiO_2 NPs (Table S1); TEM micrographs and dissolution results for Ag-B NPs (Figure S1); Hydrodynamic size and ζ-potential of Ag NPs obtained from NanoComposix (Figure S2); Putative organic surface layer on Ag-B NPs (Figure S3); CD spectroscopy of S-protein and NPs (Figure S4); CD spectroscopy (negative controls) (Figure S5); FT-IR of S1/S2 peptides and Ag-B NPs (Figure S6); FT-IR results (negative controls) (Figure S7); NRF2 induction in BEAS-2B cells (Figure S8); Cytotoxicity assessment of Ag NPs *versus* soluble salt in primary cells (HNEC) (Figure S9); Cytotoxicity assessment of TiO_2 NPs in primary cells (HNEC) (Figure S10).

Table S1. Physicochemical characterization of Ag NPs and TiO_2 NPs in various media.†

Exposure medium	0 h		24 h	
	Ag NPs	TiO_2 NPs	Ag NPs	TiO_2 NPs
Hydrodynamic diameter (d.nm)				
dH_2O (pH 7.4)	347 ± 35	680 ± 25	121 ± 28	292 ± 6
Tris-HCl (pH 7.2)	249 ± 45	878 ± 11	139 ± 33	2199 ± 513
NECM (pH 7.4)	345 ± 35	555 ± 9	211 ± 17	277 ± 0.6
DMEM (pH 7.4)	245 ± 40	392 ± 48	115 ± 9	184 ± 4
Surface charge/ζ-potential (mV)				
dH_2O (pH 7.4)	-44 ± 2	22 ± 0.8	-33 ± 1,5	23 ± 0.4
Tris-HCl (pH 7.2)	-33 ± 1.5	- 14 ± 0.9	-32 ± 1.5	- 13 ± 0.4
NECM (pH 7.4)	-11 ± 0.3	-10 ± 0.4	-11 ± 1.2	-10.5 ± 1.2
DMEM (pH 7.4)	-11.4 ± 1	-11.7 ± 0.8	-11.2 ± 0.5	-11.2 ± 1.3

†Tris-HCl buffer (10 mM) was used for CD spectroscopy measurements of NP interactions with the S-protein; NECM is the growth medium used for primary nasal epithelial cells (HNEC) (cytotoxicity assays: Alamar blue, LDH release); DMEM supplemented with 10% fetal bovine serum (FBS) is the medium used for HEK293T-ACE2 and H1299-ACE2-TMPRRS2 cells.

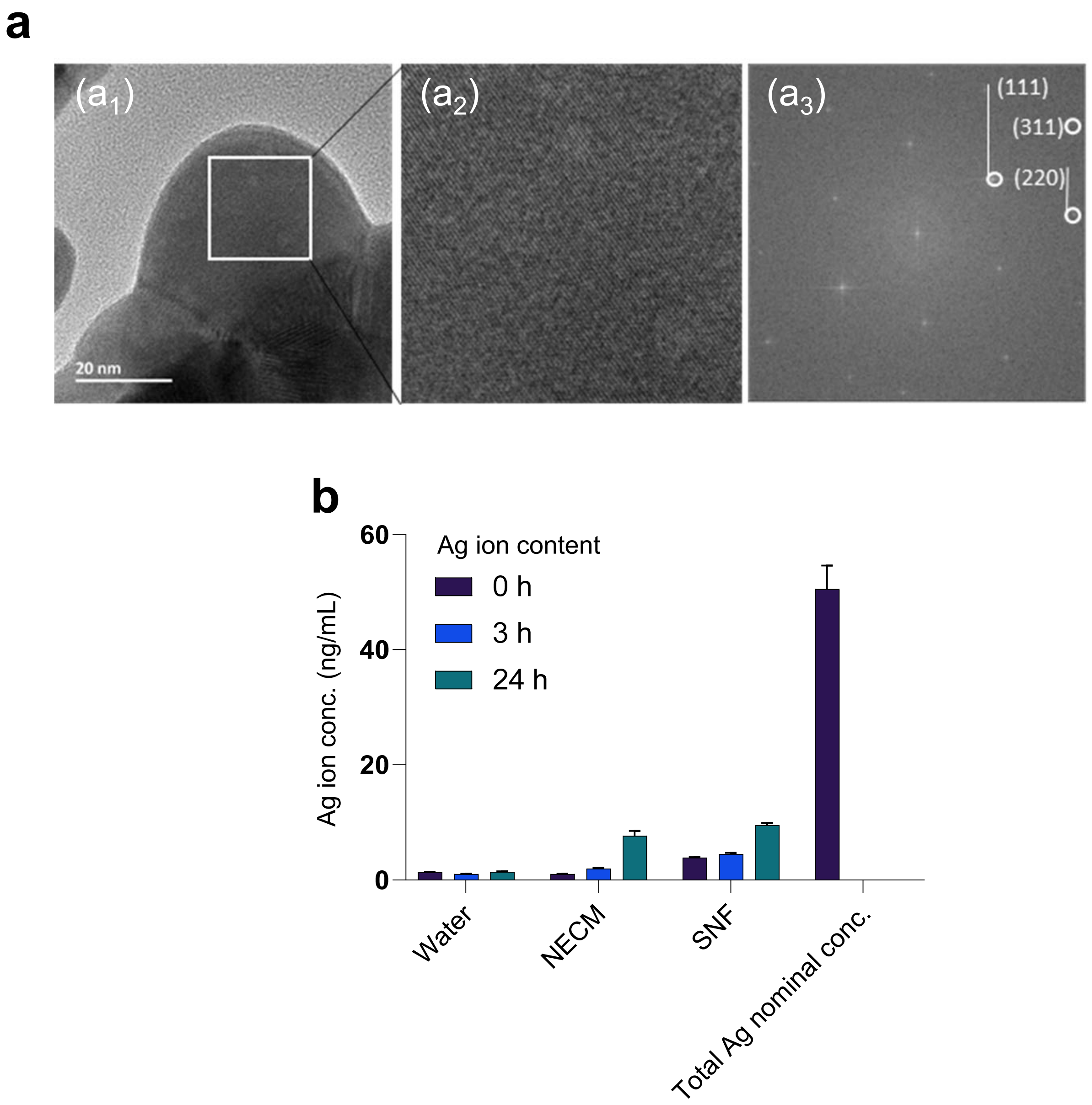

Figure S1. Characterization of Ag-B NPs procured from Sigma. (a) TEM micrographs and FFT of Ag-B NPs (a_1–a_3). Panel (a_2) shows lattice fringes, and a few points in (a_3) are matched to cubic silver (ICDD card no: 00-001-1167). (b). Dissolution of Ag-B NPs was evaluated in dH_2O, NECM, and SNF (refer to Table S1 and to the main text for further details on these media). The Ag ion content was determined by ICP-MS at the indicated time-points.

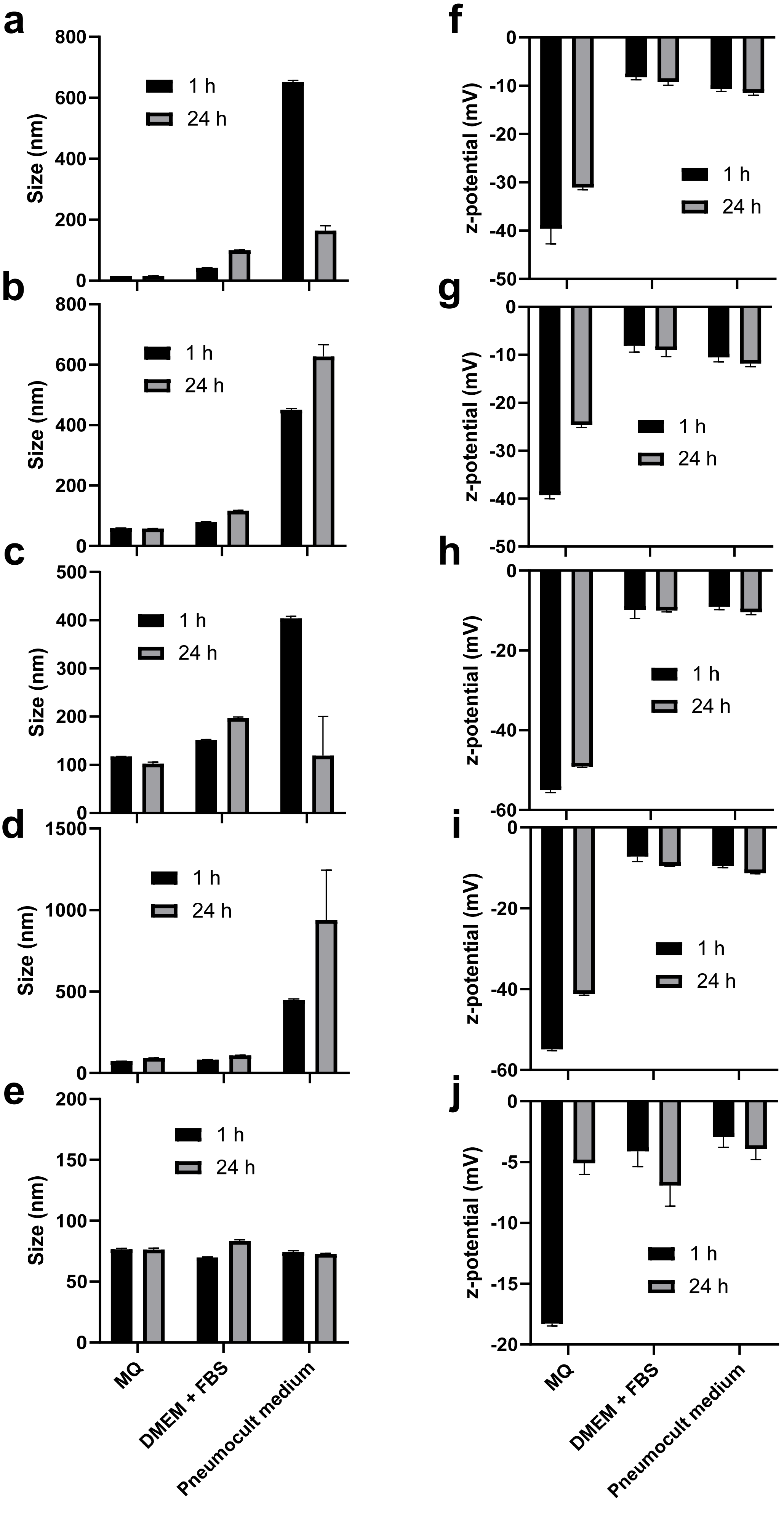

Figure S2. Characterization of Ag NPs from NanoComposix. The NPs were characterized with respect to hydrodynamic size (a-e) and surface charge (f-j) in dH_2O *versus* the cell culture medium used for the H1299 cell line (pseudovirus neutralization assay) (DMEM + 10% FBS) and for the BEAS-2B cell line (cell viability/metabolic activity assay) (Pneumacult^{TM}, no FBS supplementation). Hydrodynamic diameter at 1 h and 24 h for: (a) Ag10 (10 nm), (b) Ag50 (50 nm), (c) Ag100 (100 nm), (d) PVP-Ag50, (e) PEG-Ag50 NPs. ζ-potential values at 1 h and 24 h for: (f) Ag10 (10 nm), (g) Ag50 (50 nm), (h) Ag100 (100 nm), (i) PVP-Ag50, (j) PEG-Ag50 NPs.

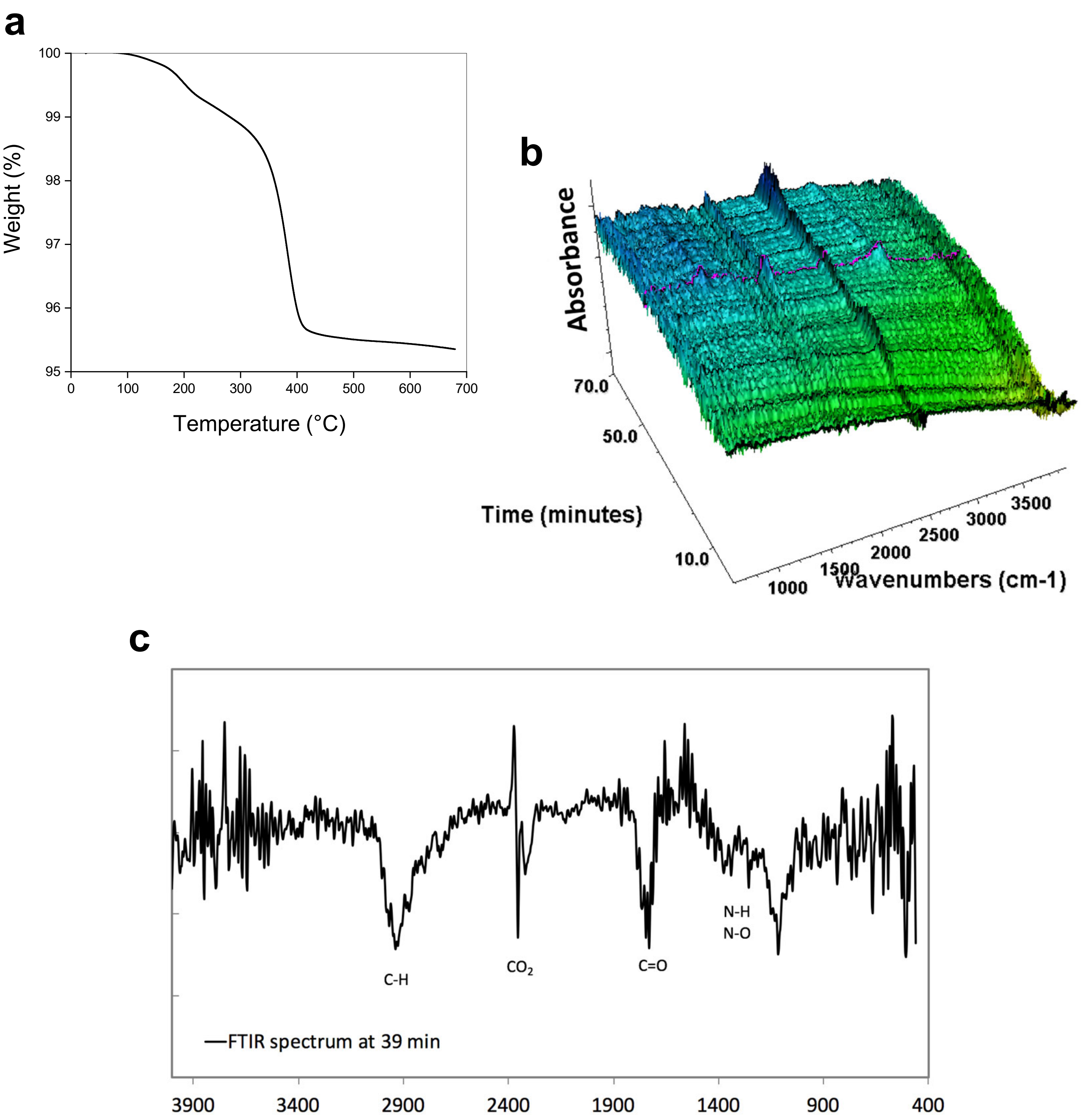

Figure S3. Tentative assignment of organic coating on Ag-B NPs from Sigma. (a) TGA thermogram of the Ag-B NPs showing a 4.5% weight loss, possibly due to an organic capping agent. (b) Evolved-gas analysis (EGA) of the Ag-B NPs. The resultant EGA spectra are shown, where the time axis in the EGA plot corresponds to the time/temperature axis in the TGA thermogram. (c) FTIR transmission spectrum corresponding to the purple line in panel (b). The observed bands are attributed to the presence of C-H, CO_2, C=O and ether groups, which are typical degradation products of carbohydrates. The region attributed to N-H and N-O could result from the reaction of the inert carrier gas N_2.

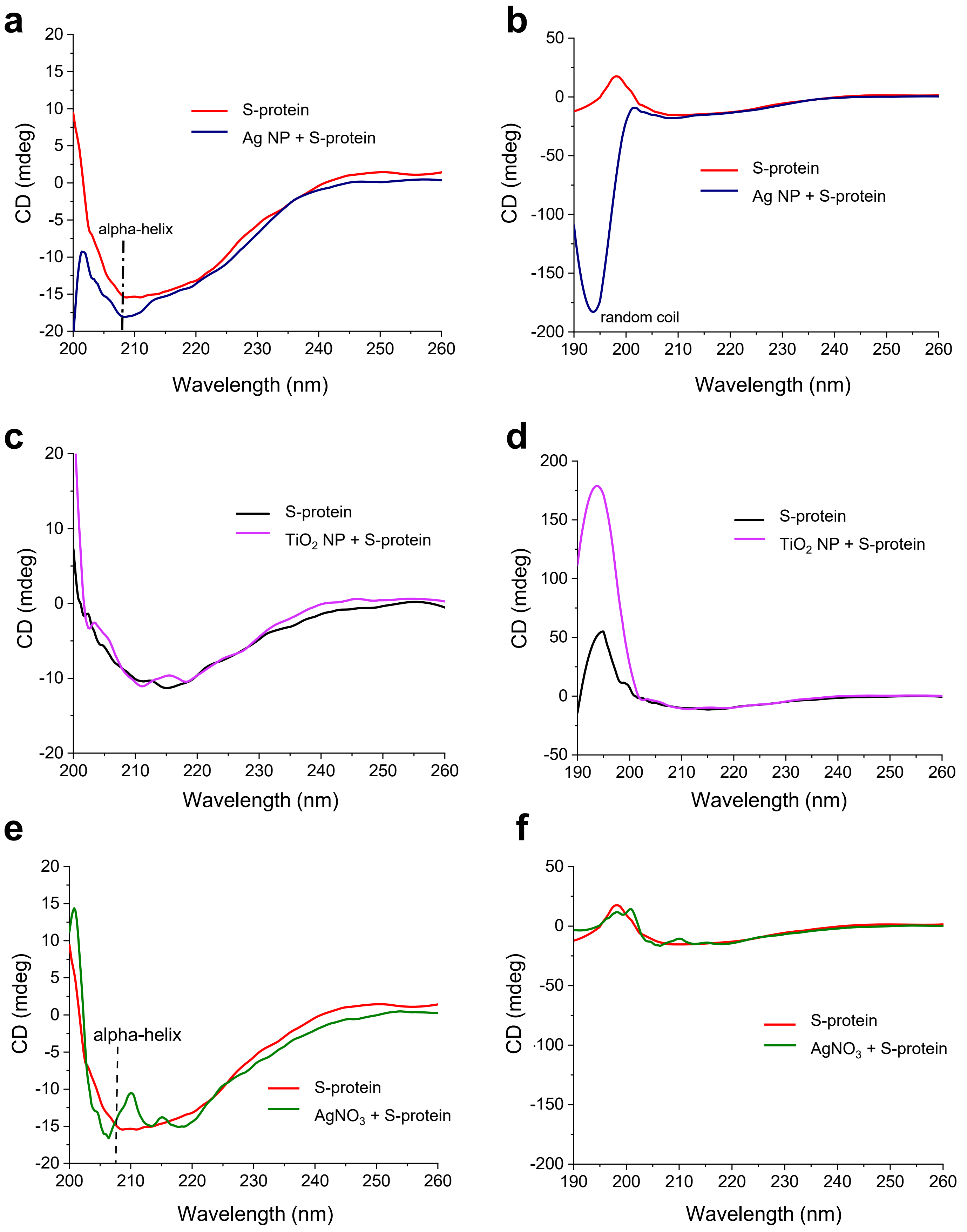

Figure S4. Ag NPs altered the α-helical content of the S-protein. (a) CD spectra obtained at 200-260 nm showed changes in the secondary structure of the S-protein (20 μg/mL) after incubation for 1 h with Ag-B NPs. (b) CD spectra showing the formation of random coils at 195 nm after interaction of the S-protein with Ag-B NPs. TiO_2 NPs had no impact on the α-helical content of the S-protein (c), and no random coils (195 nm) were observed in the presence of TiO_2 NPs (d). Similarly, $AgNO_3$ had no impact on the α-helical content (e), and no random coils were observed (f).

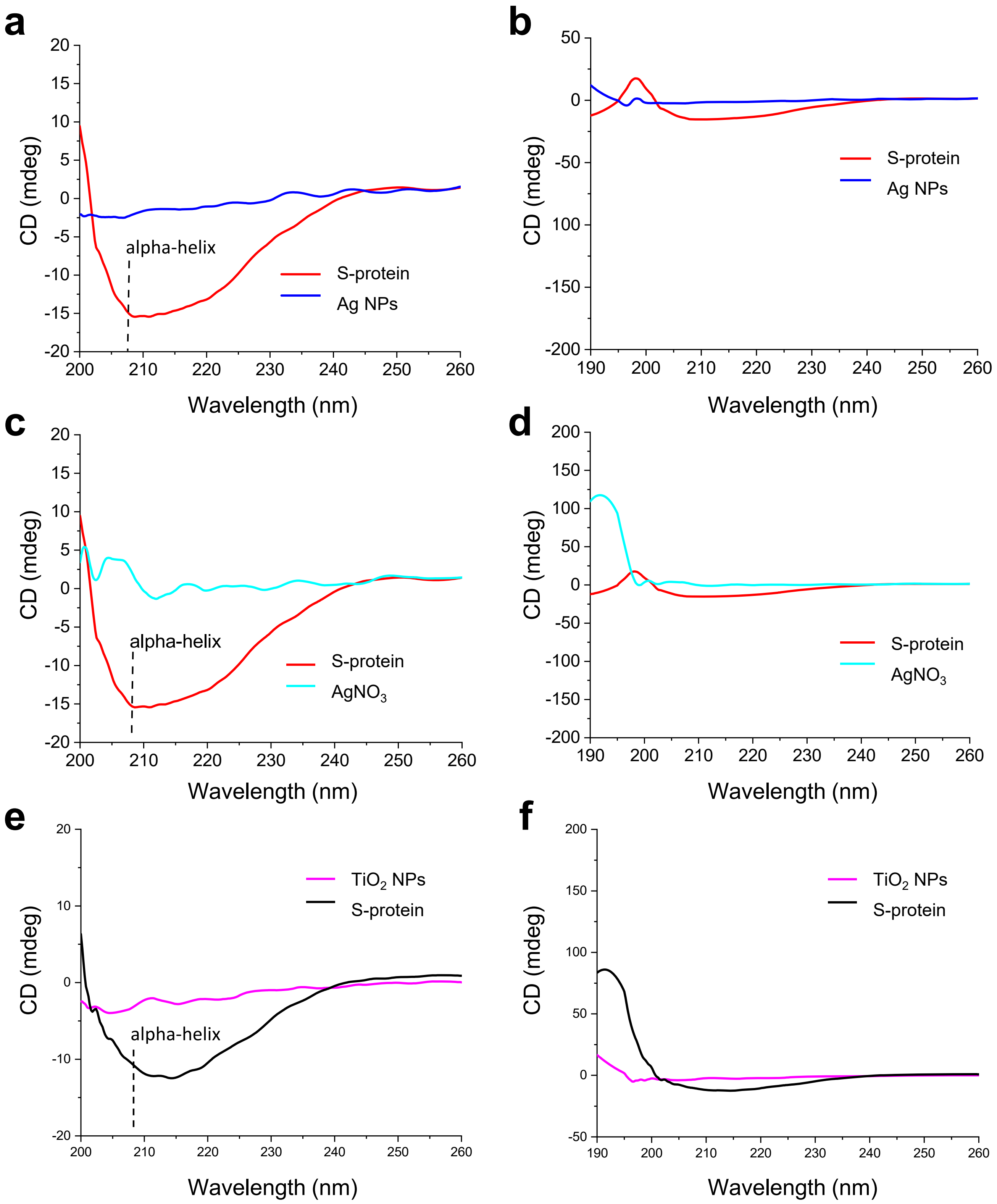

Figure S5. The tested NPs did not interfere with CD spectroscopy measurements. (a,b) Ag NPs, (c,d) $AgNO_3$ and (e,f) TiO_2 NPs 10 µg/mL were evaluated alone *versus* S-protein alone to assess whether the NPs or the salt interfered with CD measurements (cf. Figure S4).

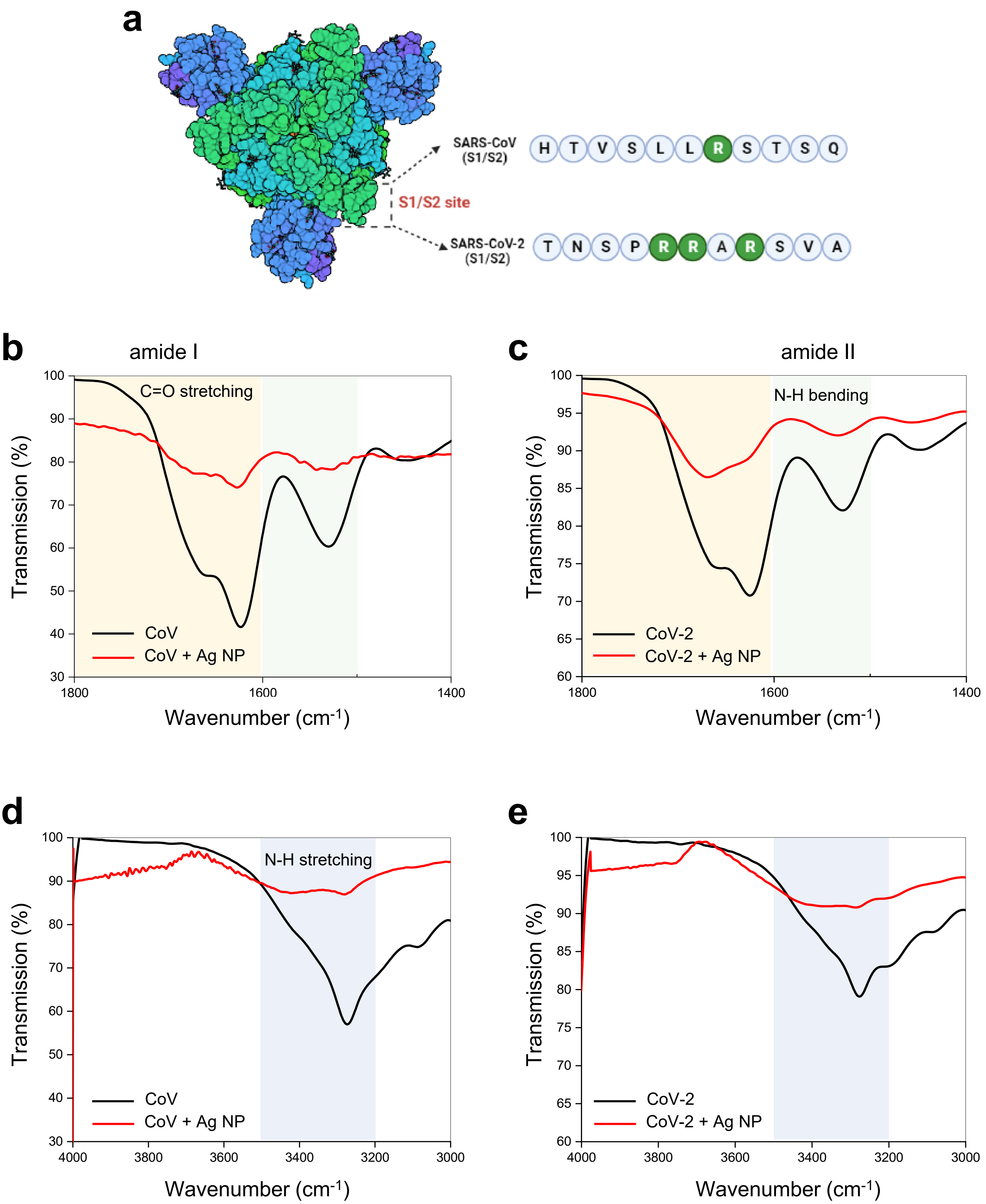

Figure S6. Interaction of the bare Ag NPs (Ag-B) with S1/S2 peptides. (a) The amino acid sequence of the SARS-CoV and SARS-CoV-2 peptides representing the proteolytic cleavage site at the S1/S2 junction of the S-protein. The figure was prepared in BioRender using S-protein coordinates derived from Xiong et al. (2020) archived at the Protein Data Bank (PDB no: 6ZP2). (b-e) FT-IR spectra of SARS-CoV (a,c) and SARS-CoV-2 peptides (b,d) in comparison to spectra in the presence of Ag-B NPs.

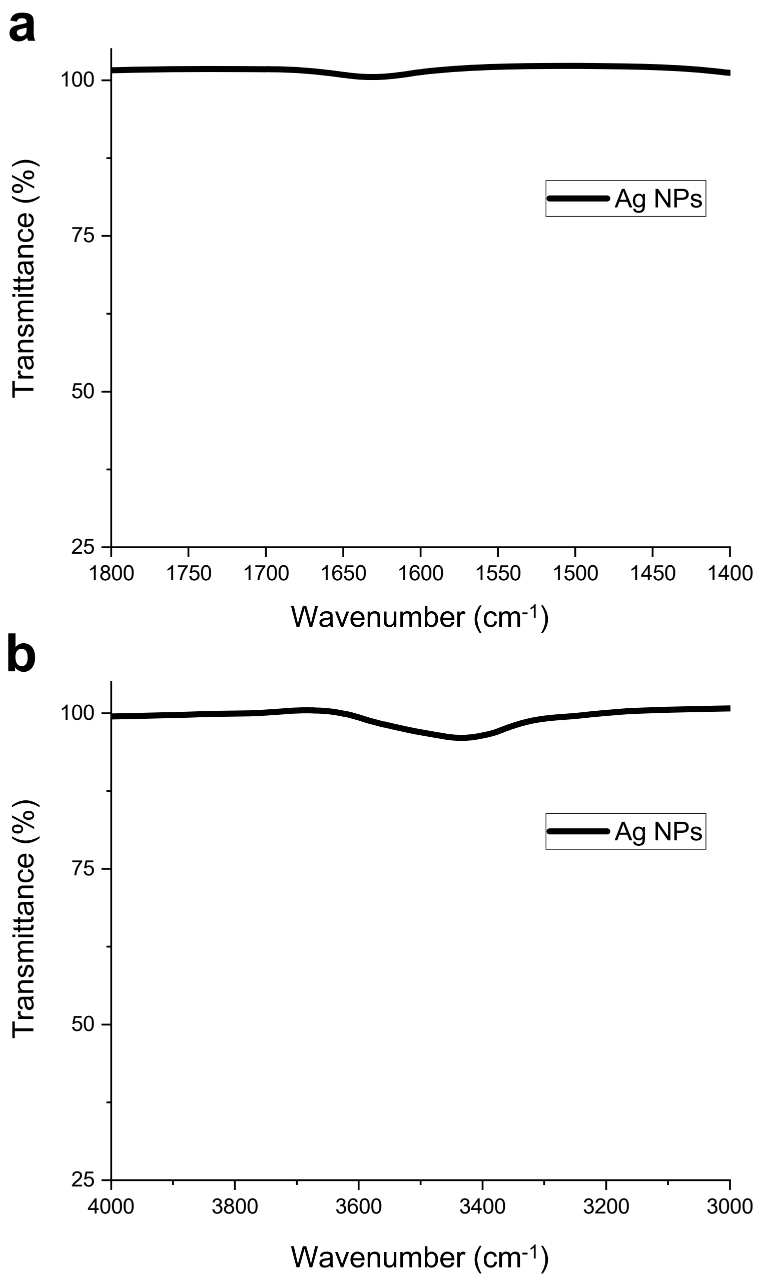

Figure S7. The tested NPs did not interfere with FT-IR measurements. FT-IR spectra of Ag-B NPs alone at regions (a) 1800-1400 cm^{-1} and (b) 4000 – 3000 cm^{-1} (refer to Figure S6).

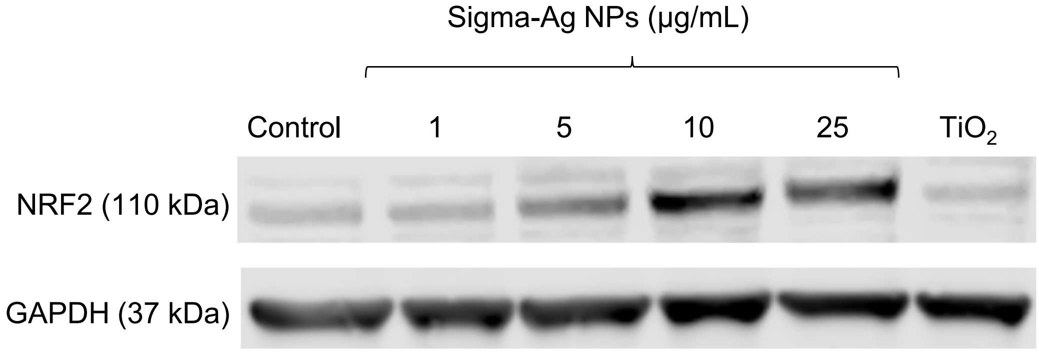

Figure S8. Dose-dependent activation of NRF2 in BEAS-2B cells determined by western blot at 24 h of exposure to Ag NPs (Sigma). TiO_2 NPs (25 µg/mL) were included as a reference. The membrane was reprobed with antibodies against GAPDH as a loading control.

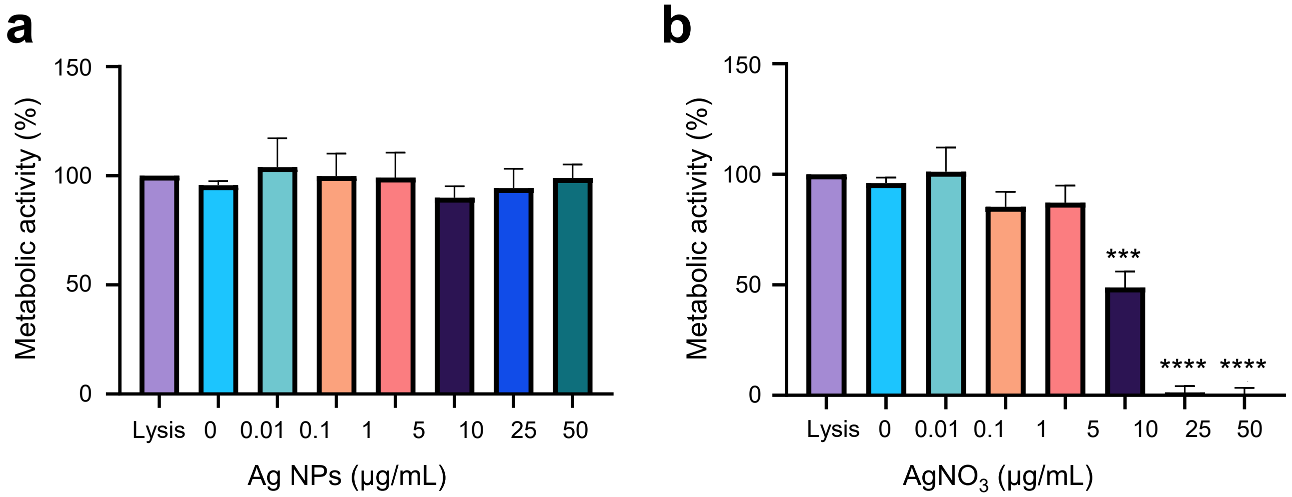

Figure S9. Cytotoxicity assessment of Ag NPs. Cell viability (metabolic capacity) of primary human nasal epithelial cells (HNEC) maintained in serum-free NECM was evaluated using the Alamar blue assay following exposure to Ag-B NPs (a) *versus* $AgNO_3$ (b). HNEC were exposed at the indicated concentrations of NPs or the soluble salt for 24 h. Data are mean values ± S.D. (n=3). ***$p<0.001$, ****$p<0.0001$. Results from the LDH assay are shown in Figure 6.

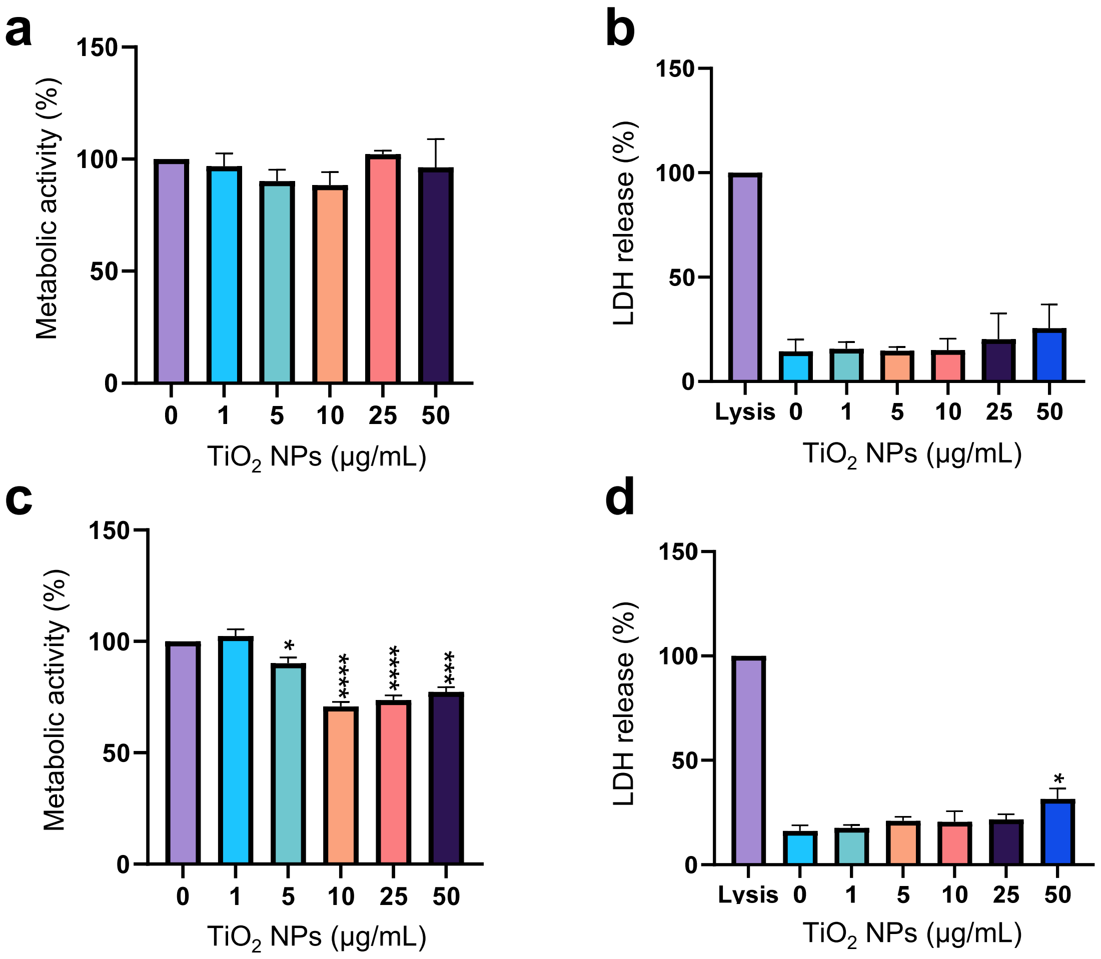

Figure S10. Cytotoxicity assessment of TiO_2 NPs. Primary human nasal epithelial cells were exposed for 24 h (a,b) or 48 h (c,d). Cell viability was evaluated using the Alamar blue assay (a,c) and the LDH release assay (b,d). Data shown are mean values ± S.D. of three independent experiments. *$p<0.05$, ***$p<0.001$, ****$p<0.0001$ (one-way ANOVA).
